# Supplementary material for: Decoding Protein–Membrane Binding Interfaces from Surface-Fingerprint-Based Geometric Deep Learning and Molecular Dynamics Simulations
Source: J Chem Inf Model. 2026 Feb 2;66(4):2299–310. doi: 10.1021/acs.jcim.5c02566 (PMC12933713; doi:10.1021/acs.jcim.5c02566)
Supplement: Supplementary file 1 [file ci5c02566_si_001.pdf]

Supporting Information

Decoding protein–membrane binding interfaces from surface-fingerprint-based geometric deep learning and molecular dynamics simulations

ByungUk Park[1] and Reid C. Van Lehn[1,2,*]

[1]Department of Chemical and Biological Engineering and [2]Department of Chemistry

University of Wisconsin−Madison, Madison, Wisconsin 53706, United States

*address correspondence to: vanlehn@wisc.edu

Table of Contents

PDBs excluded from training and test sets 2

Train–test split rationale 5

Data preprocessing 6

Surface feature calculations 7

Geometric deep learning on protein surface using learned soft polar grid 8

  Model architecture 9

Evaluation of model structure for MaSIF-PMP 10

Model performance for different superfamilies 15

Comparison with other models 17

  Mapping surface-level predictions to residue-level 17

  Metrics for comparison 18

Binary predictions of MaSIF-PMP using optimal thresholds 22

Analysis of surface features for IBS vs. non-IBS patches 27

Transfer learning of PPI-trained model to IBS predictions 30

Data augmentation using MD simulations of PMPs in aqueous solution 32

Ensemble learning with MaSIF-PMP and MD simulation data 33

Case studies using HMMM simulations 35

  α-tocopherol transfer protein (α-TTP) 35

  Defining consensus and union IBS label based on trajectories of replica simulations 37

  Oxysterol-binding protein homologue (Osh4) 40

  Representative HMMM membrane types: anionic and zwitterionic 43

PDBs excluded from training and test sets

The PMP dataset[1] published in 2022 originally comprised 1,328 domain structures with residue-level annotations of membrane-binding and non-binding interfaces. Some entries shared the same protein chain structure but were annotated as distinct experimental structures with different CATH identifiers (Supporting Fig. 1).[2] When counting unique protein structures based on PDB and chain identifiers, the dataset comprises 1,199 distinct entries. For cases in which multiple experimental structures shared the same RCSB monomer structure, we integrated their interface labels to generate a single set of labels for the corresponding PDB and chain IDs. Of 1,199 distinct single-chain structures, 10 proteins were excluded from both the training and test sets, resulting in a final dataset of 1,189 proteins. Seven of these ten proteins were removed due to extensive unmodeled regions in the central part of their sequences, which led to poorly defined molecular surface representations. The remaining three proteins were excluded because they lacked true interface annotations entirely. The corresponding PDB and chain identifiers are listed in Supporting Table 1.

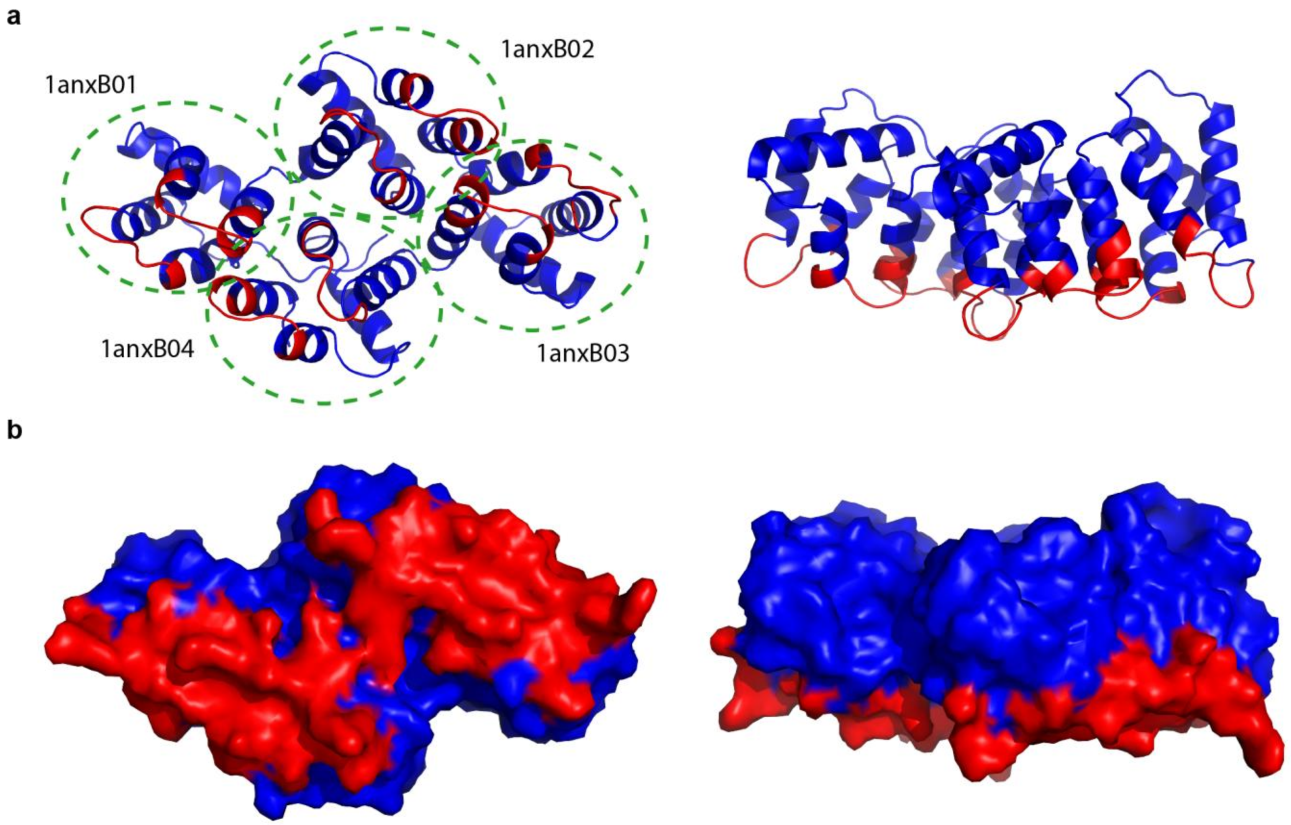

Supporting Figure 1. Illustration of the redundancy of single-chain protein structures in the dataset. Representative snapshots of annexin V (PDB ID: 1ANX, chain B) from bottom- and side-views are shown in **(a)** ribbon and **(b)** surface representations. IBS-labeled residues and surfaces are colored red, whereas non-IBS regions are colored blue. Green dashed ellipses indicate experimental structures counted as distinct entries in the dataset with different CATH identifiers (*i.e.*, 1anxB01, 1anxB02, 1anxB03, 1anxB04).[2] To avoid redundancy in training, we used only a single monomer structure of these four with its corresponding IBS when training MaSIF-PMP.

Supporting Table 1. PDB and chain IDs of proteins excluded from the PMP dataset used for model training and evaluation. Each entry is listed as a PDB ID followed by its chain ID, separated by an underscore.

	**PDB_CHAIN IDs**
Unmodeled residues in the central region of the sequence	2DYB_B, 3QBV_D, 4UXJ_H, 5CCG_E, 5KJ7_K, 3M7F_B, 3TVV_B
No interface annotations	1P8J_B, 2ID4_A, 4OMD_B

Train–test split rationale

The curated dataset of 1,189 proteins was split into 1,059 training and 130 test proteins, following the same training-to-test ratio used in the original MaSIF-site study.[3] To preserve structural diversity and ensure generalizability, the split was performed such that both sets maintained similar distributions of proteins across different superfamilies. This consideration was particularly important given that the dataset's interfacial binding site (IBS) labeling strategy relies on the assumption that structurally related proteins within the same superfamily share similar membrane-binding sites. The number of proteins assigned to each superfamily in the training and test sets is detailed in Supporting Table 2.

Supporting Table 2. Distribution of proteins across superfamilies in the training and test sets. Superfamily labels correspond to membrane-targeting domains (Annexin, C1, C2, Discoidin C2, PH, PX), enzymes (PLA, PLC/D), and lipid-transfer proteins (START).

	Training set	Test set	Total
Annexin	42	5	47
C1	33	4	37
C2	90	11	101
Discoidin C2	256	32	288
PH	234	29	263
PX	41	5	46
PLA	126	15	141
PLC/D	60	7	67
START	177	22	199
Total	1059	130	1189

Data preprocessing

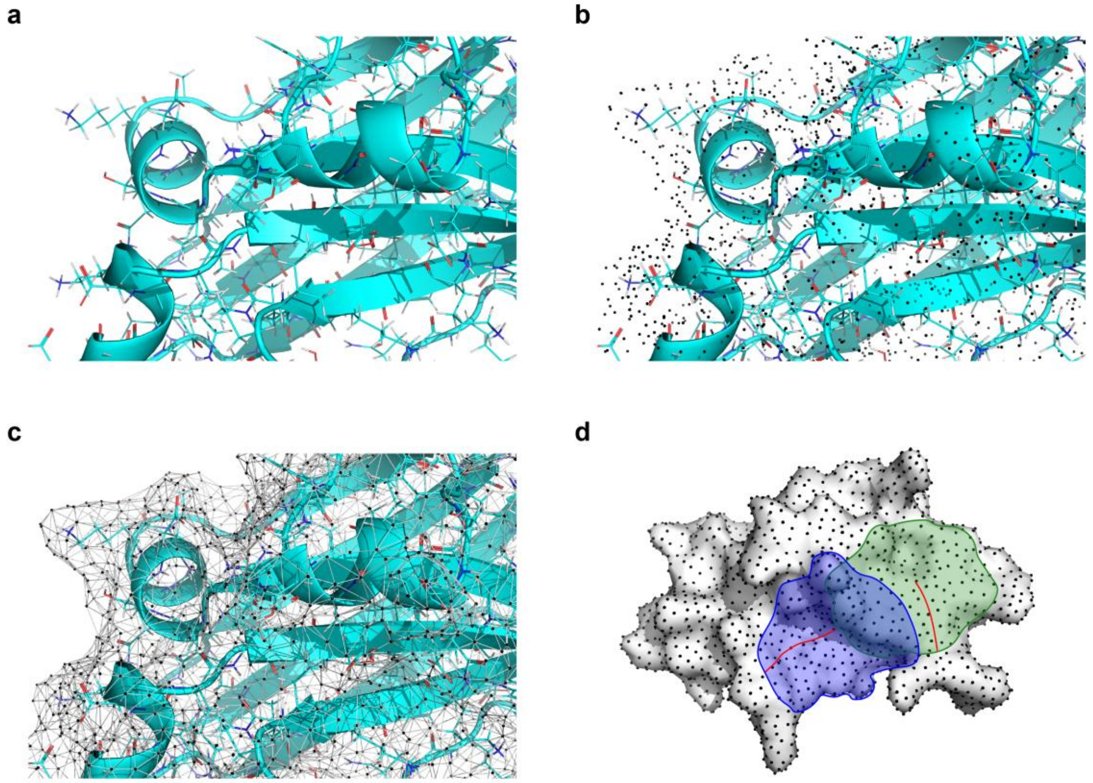

Supporting Figure 2. Illustration of the decomposition of protein surfaces into overlapping radial patches. The input protein structure **(a)** is first preprocessed to generate dot surface **(b)** using the MSMS program.[4] A graph representation **(c)** is then constructed, where vertices correspond to the surface dots and edges connect neighboring vertices. This mesh is further regularized to produce a discretized triangulated surface. Each surface vertex serves as the center of radial patch **(d)**, and neighboring vertices within a fixed geodesic radius (9 Å in MaSIF-PMP) are included in the patch. Surface features of the vertices are computed and embedded into a numerical array representing the patch-level input.

Surface feature calculations

Shape index

The shape index (Equation S1) describes the local geometry around each point on the surface based on curvature.[5] Its values range from −1 (highly concave) to +1 (highly convex) and are defined in terms of the principal curvatures κ_1 and κ_2, where $\kappa_1 \geq \kappa_2$ (Equation S2), as follows:

$$\text{shape index} = \frac{2}{\pi}\tan^{-1}\frac{\kappa_1 + \kappa_2}{\kappa_1 - \kappa_2} \tag{S1}$$

$$\kappa_1 = H + \sqrt{H^2 - K}, \kappa_2 = H - \sqrt{H^2 - K}, \tag{S2}$$

H is the vertex mean curvature and K is the vertex Gausssian curvature, which were both computed using PyMESH.[6]

Distance-dependent curvature

For each vertex within an extracted patch, the distance-dependent curvature computes a value in the range [−0.7, 0.7] to capture the relationship between the surface point's distance to the patch center and the orientation of its surface normal relative to the center point. Further details on this feature are provided in Ref. 5. While the shape index, a principal curvature-based descriptor, characterizes the local geometry of each vertex across the entire protein surface, the distance-dependent curvature quantifies curvature at the patch level using the patch center as a reference. This feature has been shown to capture complementary information to the shape index.[3, 5]

Poisson-Boltzmann electrostatic potential

We used PDB2PQR[7] to prepare protein structures for electrostatic calculations, and Poisson–Boltzmann electrostatics were computed using APBS[8] (v.1.5). The electrostatic potential at each vertex of the triangulated molecular surface was assigned using Multivalue, a utility within the APBS suite.[8] Charge values exceeding +30 or falling below −30 were capped at those respective limits, after which all values were normalized to a range between −1 and 1.

Hydrogen bond potential

The locations of free electrons and potential hydrogen bond donors (proton donors) on the molecular surface were computed using a hydrogen bond potential model as described in Ref. 9. Surface vertices whose nearest atom was a polar hydrogen, nitrogen, or oxygen were identified as potential hydrogen bond donors or acceptors. A value drawn from a Gaussian-shaped potential function was then assigned to each vertex based on the orientation between the relevant heavy atoms. These values range from −1 (optimal position for a hydrogen bond acceptor) to +1 (optimal position for a hydrogen bond donor).

Hydropathy index

Each surface point was assigned a hydropathy value based on the Kyte and Doolittle scale[10], according to the amino acid identity of the closest atom. Original values, which range from −4.5 (most hydrophilic) to +4.5 (most hydrophobic), were normalized to a range between −1 and 1.

Geometric deep learning on protein surface using learned soft polar grid

Using learned soft polar grid on a molecular surface enables a generalization of the convolutional networks (CNN) paradigm to non-Euclidean manifolds.[11, 12] The learned soft polar grid used in this work contains θ angular bins and ρ radial bins for a total of $J = \rho\theta$ bins. For each vertex in the discretized molecular surface x, with neighbors $N(x)$ and each vertex $y \in N(x)$, we define the coordinates $u(x, y)$ as the radial and angular coordinates of y with respect to x. The mapping of each grid cell j for feature vector f and the patch centered at x, $D_j(x)f$, is defined as:

$$D_j(x)f = \sum_{y \in N(x)} w_j\big(u(x,y)\big)f(y)\,, j = 1, \dots, J \qquad \text{(S3)}$$

where w_j is a Gaussian weight function and $f(y)$ are the features at vertex y. In our model, 4 angular bins and 3 radial bins are used, resulting in a total of $J = 12$ Gaussian bins. To ensure rotational invariance in the neural network, we performed 4 rotations of the input patch and conducted a max-pool operation on the output.[13] Further details of using learned soft polar grid on molecular surface are described in Refs. 3, 11, 12.

Model architecture

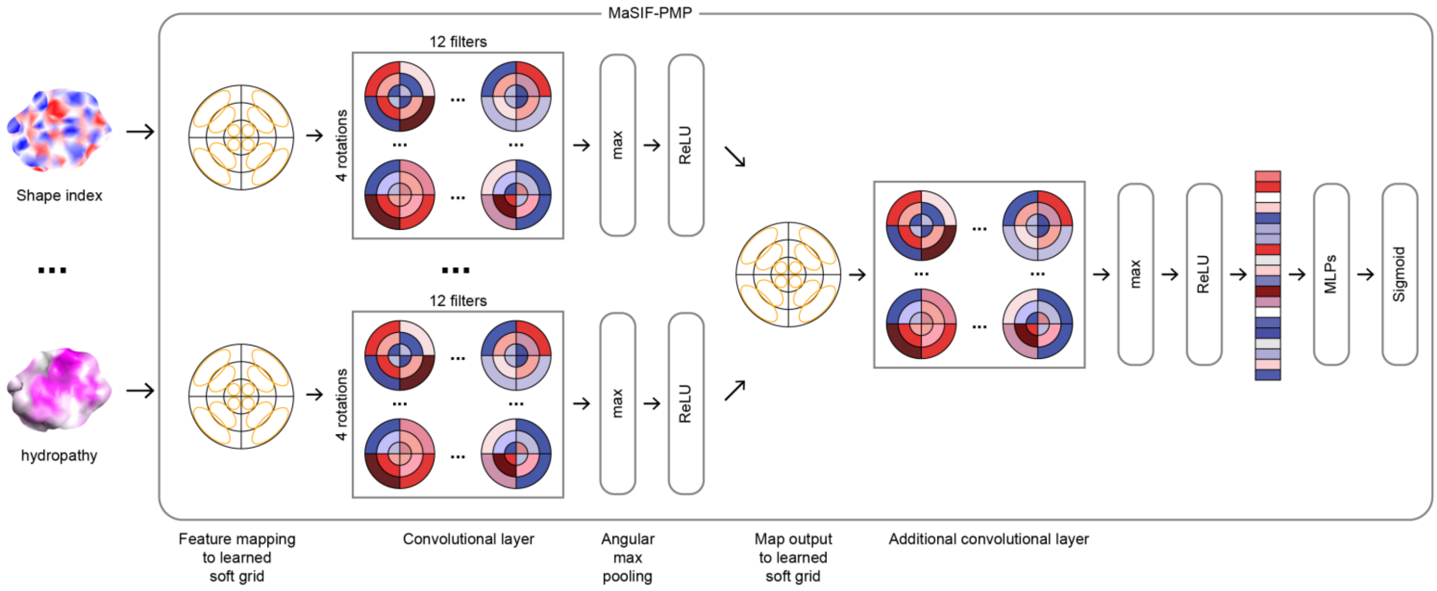

Supporting Figure 3. Network architecture of MaSIF-PMP. Surface patches are processed through a series of convolutional layers, followed by multilayer perceptron (MLP) blocks, and trained using a sigmoid cross-entropy loss function. Several elements in the figure follow similar styles as the original MaSIF publication.[3]

Evaluation of model structure for MaSIF-PMP

We evaluated the effect of incorporating layer normalization into the MaSIF-PMP architecture to improve depth stability (Supporting Fig. 4). Layer normalization standardizes feature values per surface point, stabilizing feature scales across the diverse patches and regions on the protein. Batch normalization or instance-wise spatial normalization across points may not be adequate as each point is semantically independent and the surface point number (*i.e.*, batch size) varies depending on proteins. While layer normalization increased model stability, its inclusion led to slightly lower prediction performance compared to models without it. In particular, performance decreased when layer normalization was applied to architectures with more than five convolutional layers. For models without layer normalization, instability emerged when the number of convolutional layers exceeded five. The highest prediction performance was achieved with a five-layer architecture without layer normalization.

We finalized the hyperparameters for the soft polar grid through a systematic evaluation of alternative configurations. We began with the hyperparameters used in the original MaSIF publication[3], which employed three radial and four angular bins. While the original study suggested that larger soft polar grids with increased radial and angular resolution could potentially improve training and prediction accuracy, such configurations were consistently limited by memory requirements, even when the overall grid size was kept constant. Our model achieved its highest prediction accuracy with three radial and four angular bins, whereas training with grids incorporating additional bins (*e.g.*, four or five radial bins and five or six angular bins) was constrained by memory limitations.

We also tested whether having more convolutional layers might lead to overfitting of the model by comparing two networks with different number of convolutional layers (Supporting Fig. 5). Based on the distribution of predicted IBS scores of all surface points from test set, we observed that the network with five convolutional layers was better at classifying true negatives (non-interface points) but shows flat distributions of scores for true positives (interface points). However, the network with three convolutional layers outputted more predictions with high interface scores, indicating that it is better for distinguishing IBS from non-IBS regions despite the lower prediction accuracy compared to the model with five convolutional

layers. We thus chose the network with three convolution layers, yielding the same structure as previous work on predicting protein-protein interactions (PPI) using MaSIF-site.[3]

Given that (i) depth-induced instability was not a critical issue at this configuration, (ii) the original MaSIF-site model for PPI prediction[3] was also implemented with three convolutional layers and without layer normalization, and (iii) the network with three convolutional layers yielded comparable prediction accuracy to the one with five layers (best-performing one) while better at predicting true binding interfaces, we selected the three-layer, no–layer-normalization architecture as the final model used for comparisons in the main text. Nonetheless, layer normalization may become necessary if the number of surface features used as input descriptors is substantially increased in future work.

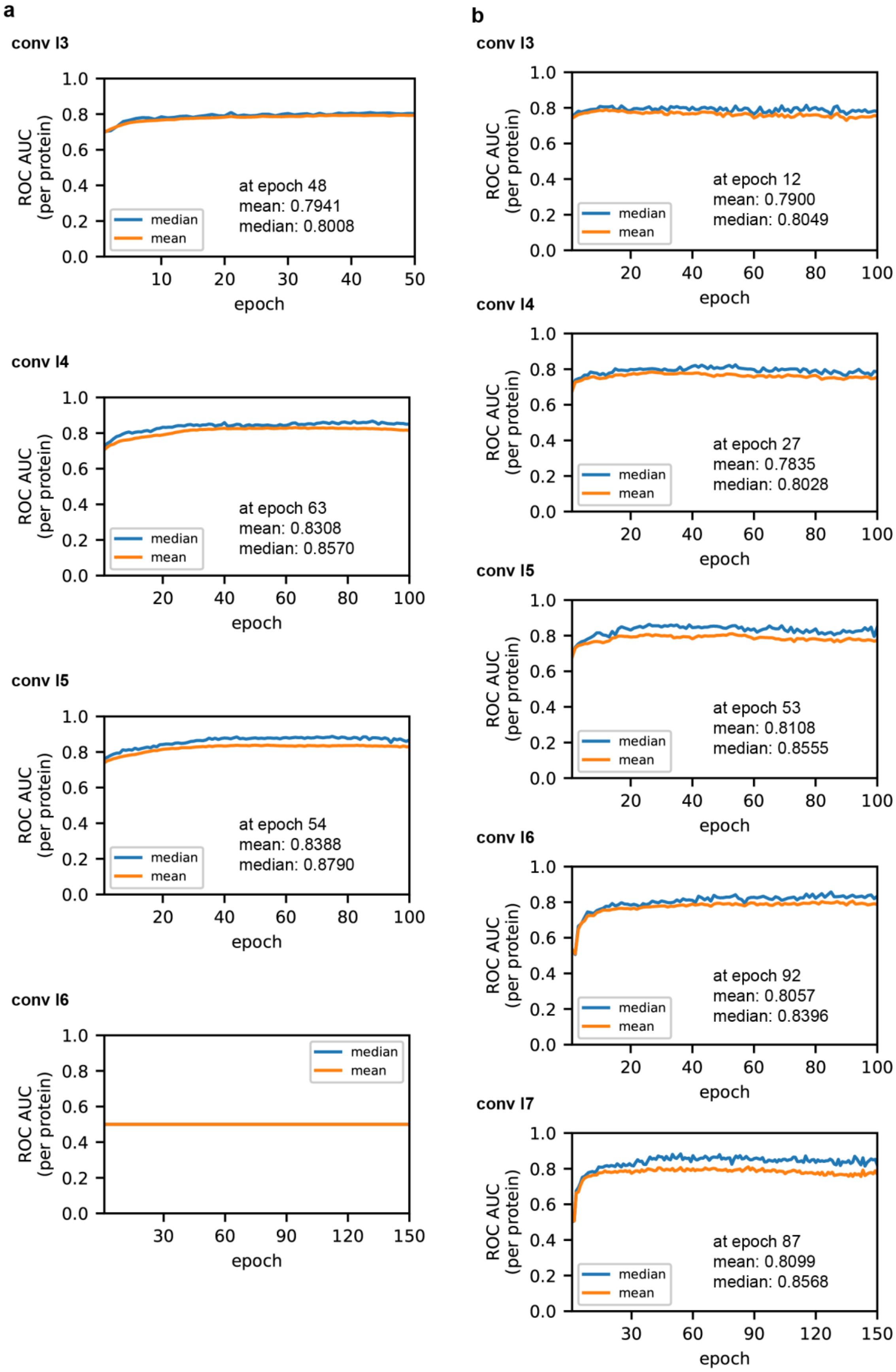
a
conv l3
ROC AUC (per protein)
epoch
median
mean
at epoch 48
mean: 0.7941
median: 0.8008
conv l4
at epoch 63
mean: 0.8308
median: 0.8570
conv l5
at epoch 54
mean: 0.8388
median: 0.8790
conv l6
b
conv l3
at epoch 12
mean: 0.7900
median: 0.8049
conv l4
at epoch 27
mean: 0.7835
median: 0.8028
conv l5
at epoch 53
mean: 0.8108
median: 0.8555
conv l6
at epoch 92
mean: 0.8057
median: 0.8396
conv l7
at epoch 87
mean: 0.8099
median: 0.8568

Supporting Figure 4. Systematic evaluation of the number of convolutional layers (noted as “conv l#”) of MaSIF-PMP. Mean and median of per protein ROC AUC scores for validation set were plotted as functions of epoch number. **(a)** Evaluations using models without layer normalization. The best model performance was observed when it contained five convolutional layers, while more layers introduced a depth-induced instability that led to a flat ROC AUC of 0.5 for all epochs. All tested models reached plateau in mean of per protein ROC AUC values within the tested epoch number. The inset text indicates the epoch number at which the best performing model was saved and the corresponding mean and median per-protein ROC AUC values. **(b)** Evaluations using models with layer normalization. Incorporating layer normalization addressed the depth-induced instability and model prediction performance decreased as the model used more than five convolutional layers.

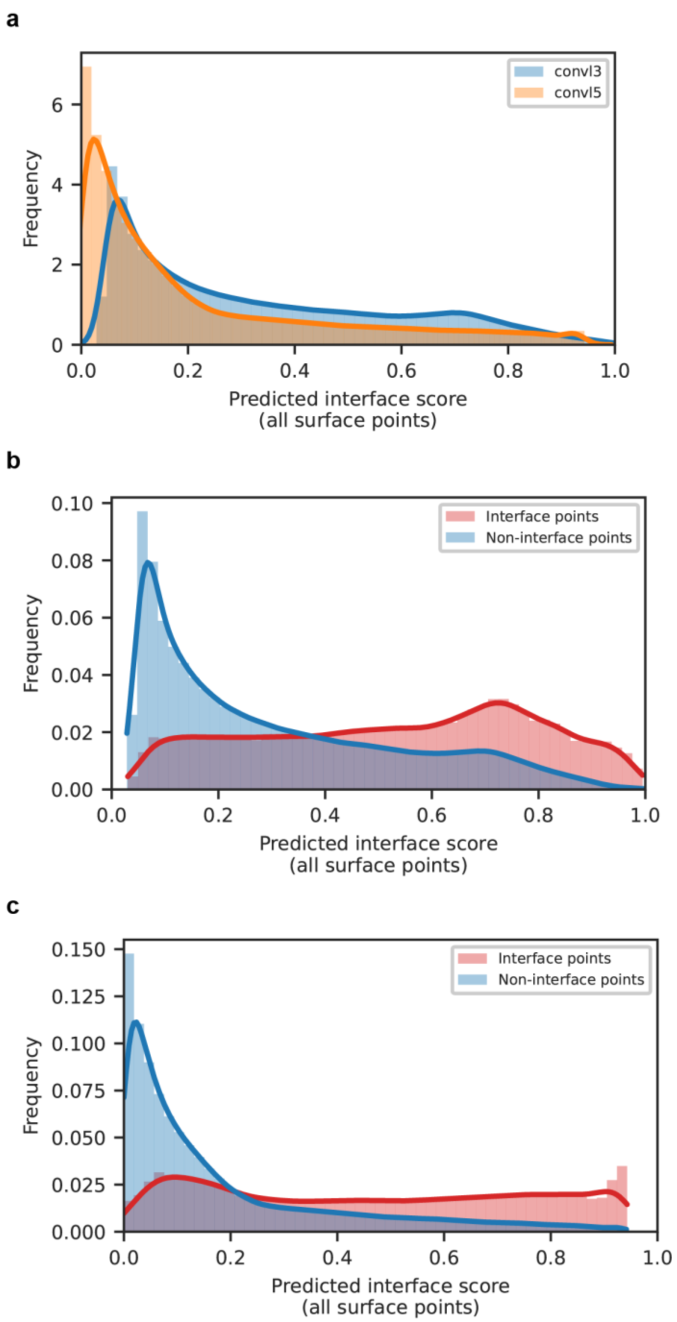

Supporting Figure 5. Distribution of predicted interface scores for the test set using models with different number of convolutional layers. **(a)** Distribution of predicted scores for all surface points in the test set for different models. “convl3” indicates the model with three convolutional layers while “convl5” indicates the model with five layers. **(b-c)** Distribution of predicted interface scores of test set for true positives (red) versus true negatives (blue) for a network trained with all features. Prediction results from model trained with **(b)** three convolutional layers and **(c)** five convolutional layers.

Model performance for different superfamilies

To perform model testing on PMPs with no homologous superfamilies present in the training set, we conducted additional training and testing of the model with an alternative strategy for generating the training and test sets. Of the nine superfamilies present in our dataset (Supporting Table 2), we selected three superfamilies that were the least represented in terms of the number of structures in the dataset: Annexin (47 structures), C1 (37 structures), and PX (46 structures). We focused on these superfamilies because they had a sufficient number of structures to test model performance on an unseen superfamily while retaining enough structures in the remaining superfamilies to permit model training. We re-trained the model three times in which we held-out all of the structures in one of these three superfamilies while training on all structures in the other eight superfamilies. For robustness, we performed training and testing three times for each split strategy where 10% of the training data (*i.e.*, 10% of the data from the eight superfamilies included in the training set) was randomly sampled and set aside as a validation set for each replica to match the training procedure described in the main text. The median ROC AUCs computed from predictions of the models tested on each family are provided Supporting Table 3. ROC AUC values for the three families (0.65 ± 0.02 for Annexin, 0.84 ± 0.03 for C1, and 0.76 ± 0.02 for PX) are comparable to the 0.78 median ROC AUC obtained using the train/test split procedure described above. Therefore, this additional test indicates that there is marginal potential inflation of test set performance due to the class-based splitting procedure used in the main text.

We also checked if any of the superfamilies, when represented in the test set using the class-based splitting procedure used in the main text, had a notably poor performance. Supporting Table 4 reports the average ROC AUC for each superfamily, which was computed by averaging the ROC AUC values across all test set structures belonging to that superfamily. No clear relationship between prediction accuracy and the number of structures in each of the homologous superfamilies is observed. We also assessed whether any superfamily had a disproportionate number of poorly predicted PMPs, which we assessed by counting the number of predicted PMPs in each superfamily that were among the bottom-25 average ROC AUC values. This analysis suggests that the PH and Discoidin C2 superfamilies may be regarded as the ones that yield the poorest prediction performance by having the most poorly predicted structures, but they are also the most abundant superfamilies in the dataset. Therefore, the proportion of superfamilies with poorly

predicted PMPs in the test set can be attributed to their proportion in the original dataset and not to the inflation on the training due to imbalance in the dataset.

Supporting Table 3. Median ROC AUCs computed from models trained using an alternative leave-one-superfamily-out strategy and evaluated on each protein family. The mean and standard deviation of the median ROC AUC were calculated from three independent replicas with randomly sampled validation sets.

Split Strategy	Median ROC AUC
Original train/test split	0.78
Annexin-excluded training	0.65 ± 0.02
C1-excluded training	0.84 ± 0.03
PX-excluded training	0.76 ± 0.02

Supporting Table 4. Average ROC AUC for each superfamily in the test set. “Number of poorly predicted PMPs” reports the number in each superfamily that were among the bottom-25 average ROC AUC values.

Superfamily	Entry number in test set	Average ROC AUC	Number of poorly predicted PMPs
Discoidin C2	32	0.71 ± 0.14	10
PH	29	0.72 ± 0.13	7
START	22	0.81 ± 0.11	2
PLA	15	0.81 ± 0.10	1
C2	11	0.80 ± 0.22	3
PLC/D	7	0.76 ± 0.07	0
PX	5	0.73 ± 0.16	2
Annexin	5	0.78 ± 0.05	0
C1	4	0.82 ± 0.10	0

Comparison with other models

For the benchmark comparison, we selected three state-of-the-art IBS predictors, PMIpred[14], DREAMM[15], and PPM3[16]. The benchmark test set was selected from the PMIpred study and includes 21 proteins with experimentally resolved structures downloaded from the RCSB PDB—19 of which are from the DREAMM test set, and 2 are known lipid packing defect sensors. To ensure consistency and comparability, AlphaFold-predicted structures were excluded. Model predictions were generated using the same PDB and chain IDs as those in the PMIpred benchmark. Ground-truth labels were also taken directly from the PMIpred benchmark, except for three proteins (PDB IDs: 1JSS, 2RSG, 1LN1). For these, we used the broader IBS annotations from our dataset, which already encompassed the original PMIpred labels but included additional interface residues. We performed predictions on the benchmark set using each model's default parameters. For example, for PPM3, we used the default settings of an undefined membrane, no curvature, and an 'in' topology for the protein N-terminus. The resulting predictions for the benchmark set were consistent with those reported in the original PMIpred and DREAMM publications.[14, 15]

Mapping surface-level predictions to residue-level

To allow direct comparison with PMIpred, DREAMM, and PPM3, which provide residue-level interface predictions, we mapped MaSIF-PMP's surface-level outputs to the residue level (Supporting Fig. 6a). Since MaSIF-PMP generates predictions only at mesh points on the molecular surface, we restricted evaluation to surface-exposed residues.

We followed the same strategy used in the benchmark assessment of MaSIF-site[3], a previous model for PPI prediction, to convert surface-level predictions into residue-level scores. In particular, we used the SPPIDER definition[17] of interfacial residues, which has been extensively validated both qualitatively and quantitatively. According to this definition, interface residues are those whose solvent-excluded surface area changes by at least 5 $Å^2$ upon binding and contribute at least 4% to the total solvent-excluded interface area. We note that all calculations were performed using solvent-excluded, not solvent-accessible, surface areas for consistency with MaSIF's surface representation.

Residue-level IBS scores from MaSIF-PMP were computed by assigning each residue the maximum IBS score among all its associated surface points. Conversely, for PMIpred, DREAMM, and

PPM3, whose outputs are at the residue-level, we mapped their predictions onto the molecular surface using the same method described in the main text for defining IBS labels on surface points.

Metrics for comparison

To evaluate and compare predictor performance, we used both area under curve (AUC) of the receiver operating characteristic (ROC) curve and the Matthews correlation coefficient (MCC) as evaluation metrics. ROC AUC is a threshold-independent metric and offers a more robust assessment than basic metrics such as accuracy or precision (Supporting Fig. 6b). Specifically, given the model-predicted scores and corresponding ground-truth labels, threshold values were systematically varied from 0 to 1 to convert continuous scores into binary labels: residues with scores equal to or above the threshold were assigned positive labels and the rest were assigned negative labels. For a given threshold, a point was classified as a true positive (TP) or true negative (TN) if its predicted label matched the corresponding positive or negative ground-truth label, and as a false positive (FP) or false negative (FN) if it did not. True and false positive rates (Equation S4) were then computed for all thresholds to generate the ROC curve, and the area under this curve was used as the ROC AUC metric. ROC AUC scores range from 0 to 1, where 1 indicates a perfect model and 0.5 indicates random guessing. Higher AUC scores (*e.g.*, > 0.7) signify better model performance, demonstrating a greater ability to distinguish between positive and negative classes across all possible thresholds.

$$\text{True positive rate} = \frac{TP}{TP + FN}, \qquad \text{False positive rate} = \frac{FP}{TN + FP}, \tag{S4}$$

The MCC (defined in Equation S5) was computed due to its suitability for highly imbalanced classification tasks—such as in our case, where non-IBS regions outnumber IBS regions on protein surfaces. An MCC score ranges from –1 to +1, where +1 indicates perfect classification, 0 suggests random guessing, and –1 indicates perfectly incorrect classification. MCC has also been employed in previous benchmark comparisons of IBS predictors.[14, 15] We evaluated model performance at both the surface-level and residue-level by computing per-protein ROC AUC and MCC scores. For the benchmark comparison, since MaSIF-PMP outputs continuous prediction scores while MCC is calculated from discrete counts of

TP, TN, FP, and FN, the best MCC value for each protein was determined across all tested thresholds.

$$\mathrm{MCC} = \frac{TP \cdot TN - FP \cdot FN}{\sqrt{(TP + FP)(TP + FN)(TN + FP)(TN + FN)}} \tag{S5}$$

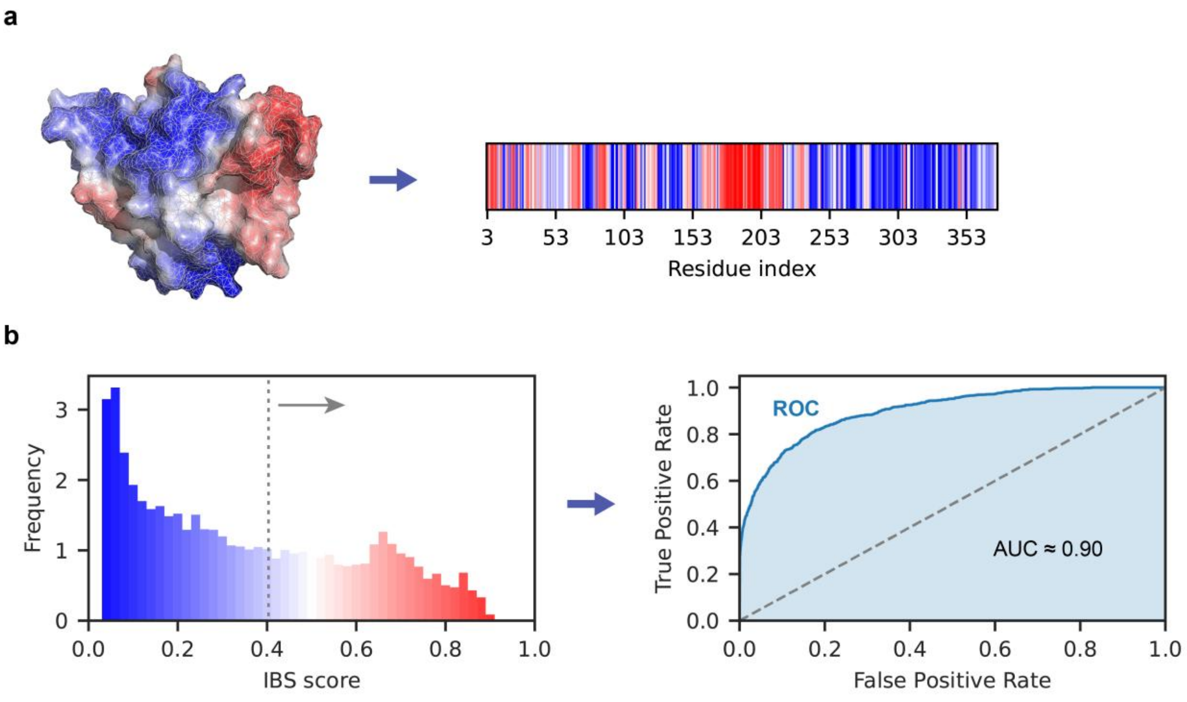

Supporting Figure 6. Benchmark comparison with IBS predictors. **(a)** Schematic illustration of the procedure used to map surface-level predictions to per-residue scores. For each residue, the highest MaSIF-PMP IBS score among its associated surface points was assigned. Snapshots shown correspond to the prediction results for (S)-mandelate dehydrogenase (PDB ID: 6BFG). **(b)** Example ROC curve generated using surface-level predictions for (S)-mandelate dehydrogenase (PDB ID: 6BFG) from the benchmark set. The distribution of surface-level IBS scores for the protein is shown, with the gray dashed line indicating the sliding threshold (0–1). Points with scores equal to or above the threshold are classified as IBSs, while those below are classified as non-IBSs. Corresponding true and false positive rates were computed across thresholds to construct the ROC curve, yielding an area under the curve (AUC) of 0.90.

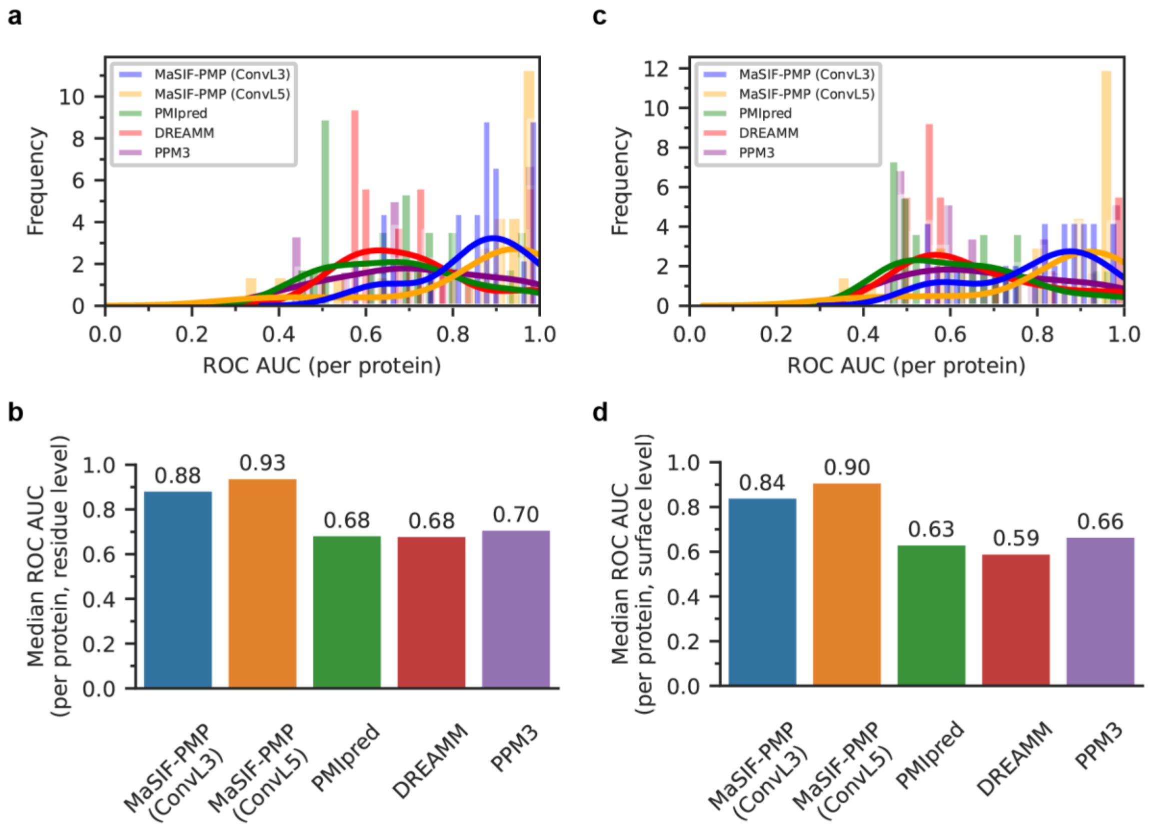

Supporting Figure 7. Benchmark comparison between different IBS predictors in terms of ROC AUC scores for 21 single-chain PMPs. For MaSIF-PMP, "ConvL3" is the network with three convolutional layers while "ConvL5" is the model with five layers. **(a)** Distribution of per-protein, residue-level ROC AUC predicted by MaSIF-PMP, PMIpred[14], DREAMM[15], and PPM3[16]. Solid lines represent Gaussian kernel density estimates fitted to the discrete score distributions. **(b)** Comparison of MaSIF-PMP with other IBS predictors on the benchmark proteins. Results are reported as the median ROC AUC per protein, evaluated on a per-residue basis. **(c–d)** Equivalent results for per-protein, surface-level ROC AUC.

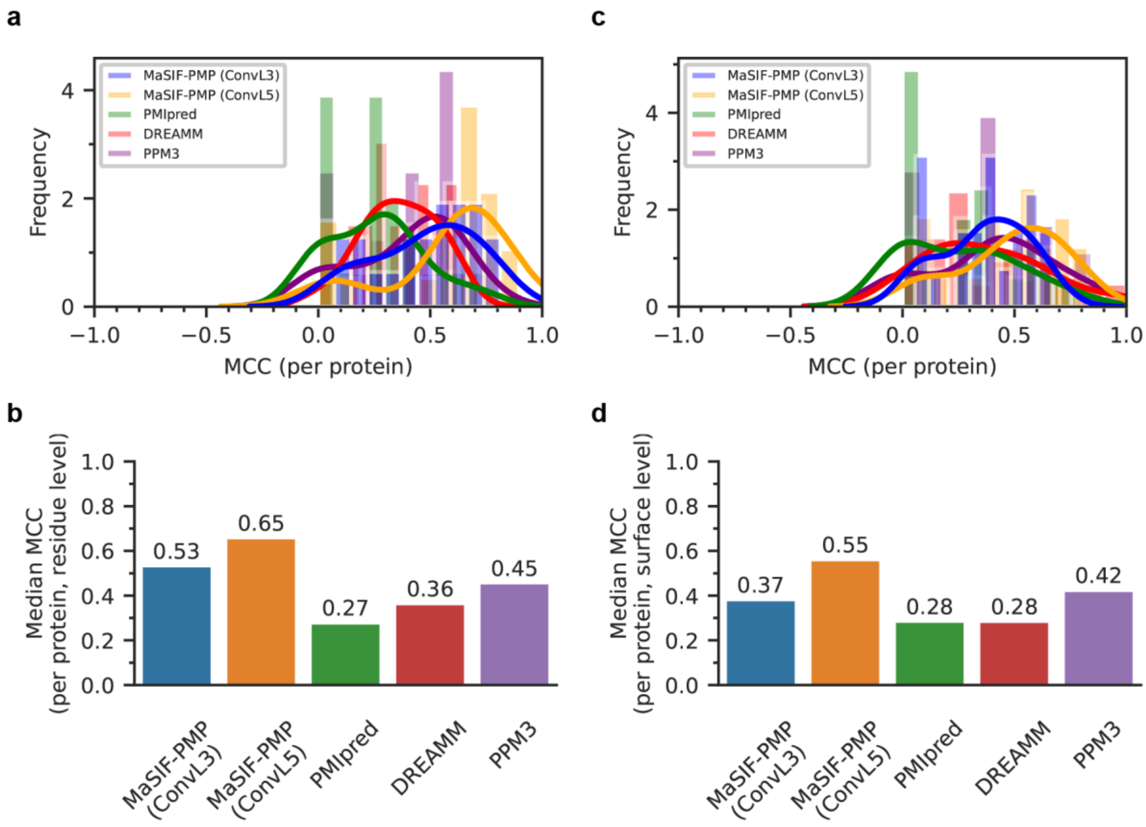

Supporting Figure 8. Benchmark comparison between different IBS predictors in terms of MCC scores. The same predictor labels as in Supporting Fig. 7 are used. **(a)** Distribution of per-protein, residue-level MCC predicted by multiple IBS predictors, with solid lines representing Gaussian kernel density estimates fitted to the discrete score distributions. For MaSIF-PMP, the best per-protein MCC was determined across all thresholds. **(b)** Comparison of MaSIF-PMP with other IBS predictors on the benchmark proteins, reported as the median per-protein MCC values evaluated on a per-residue basis. **(c–d)** Equivalent results for per-protein, surface-level MCC.

Binary predictions of MaSIF-PMP using optimal thresholds

While MaSIF-PMP outputs continuous prediction scores representing the confidence that a surface patch belongs to an IBS, binary labels—comparable to those from other IBS predictors[14-16]—can be obtained by applying a threshold. To determine the optimal thresholds for residue- and surface-level predictions, we calculated the median per-protein MCC across all training set proteins for thresholds ranging from 0 to 1 using the MaSIF-PMP model with three convolutional layers. The median MCC values were plotted as functions of the threshold values, and the optimal threshold values were determined as those yielding the maximum median MCC (Supporting Fig. 9a, 9d). Using these thresholds, MCC scores were computed for the benchmark set and visualized as score distributions (Supporting Fig. 9b, 9e) and median values (Supporting Fig. 9c, 9f). With binary predictions based on the optimal thresholds, MaSIF-PMP achieved comparable or better prediction performance than PMIpred and DREAMM, but lower performance than PPM3. Kernel density estimates of MCC exhibited right-shifted distributions for MaSIF-PMP compared to PMIpred and DREAMM at both residue and surface levels, with overall higher median MCC values. In contrast, PPM3 outperformed binary predictions from MaSIF-PMP in both MCC distributions and median values (Supporting Fig. 9b, 9c, 9e, 9f).

This behavior likely arises from MaSIF-PMP's tendency to assign high interface scores to a larger fraction of surface patches, resulting in broader regions being labeled as interfaces when thresholds are applied (Supporting Fig. 5). A similar pattern is observed in Supporting Figure 10, where MaSIF-PMP predicts more extensive interfaces than other predictors. For sphingomyelinase C (Supporting Fig. 10c), this tendency leads to lower MCC values compared to other methods, despite predicted interface regions closely matching the ground-truth labels and plausibly engaging the membrane through conformational fluctuations during binding. In contrast, Supporting Figure 8 shows that a MaSIF-PMP variant with five convolutional layers, which tends to assign lower interface scores across surface patches, substantially outperformed other predictors in both residue- and surface-level MCC. Together, these results suggest that MaSIF-PMP may be disadvantaged when evaluated using threshold-based binary labels that may not fully capture the continuous and spatially distributed nature of protein–membrane interactions.

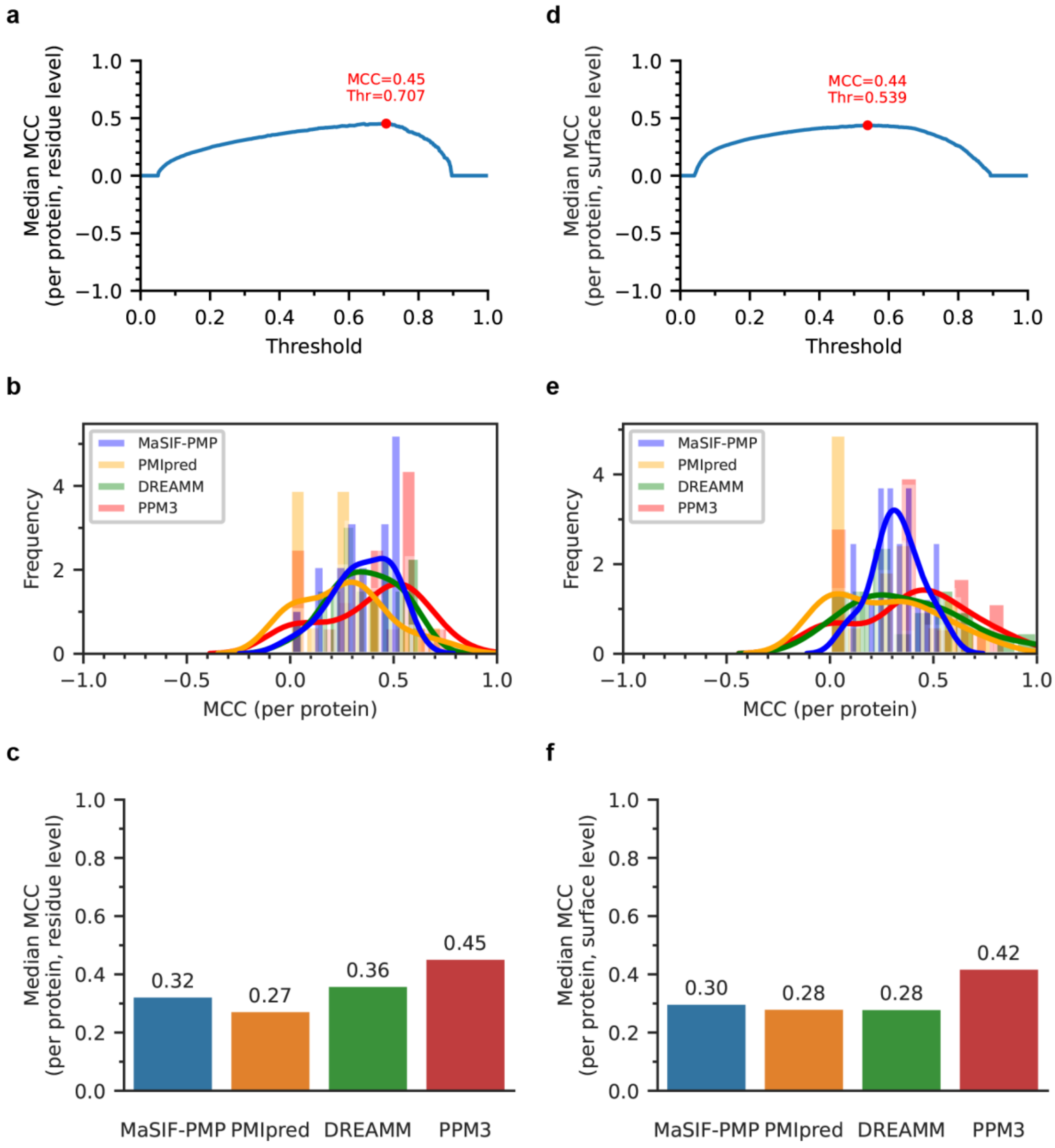

Supporting Figure 9. Applying a threshold to generate binary predictions of MaSIF-PMP with three convolutional layers. **(a)** Median per-protein, residue-level MCC as a function of threshold values. Per-protein MCC values were calculated across all residues of proteins in the training set for thresholds ranging from 0 to 1, with the maximum median MCC observed at a threshold of 0.707. **(b)** Distributions of per-protein, residue-level MCC predicted by MaSIF-PMP, PMIpred, DREAMM, and PPM3, using a threshold of 0.707 for MaSIF-PMP predictions. Solid lines represent Gaussian kernel density estimates fitted to the discrete score distributions. **(c)** Comparison of MaSIF-PMP using the same threshold with other IBS

predictors on the benchmark proteins. Results are reported as the median MCC per protein, evaluated on a per-residue basis to ensure comparability across predictors. **(d–f)** Equivalent results for per-protein, surface-level MCC. Surface-level binary predictions of MaSIF-PMP were generated using a threshold of 0.539.

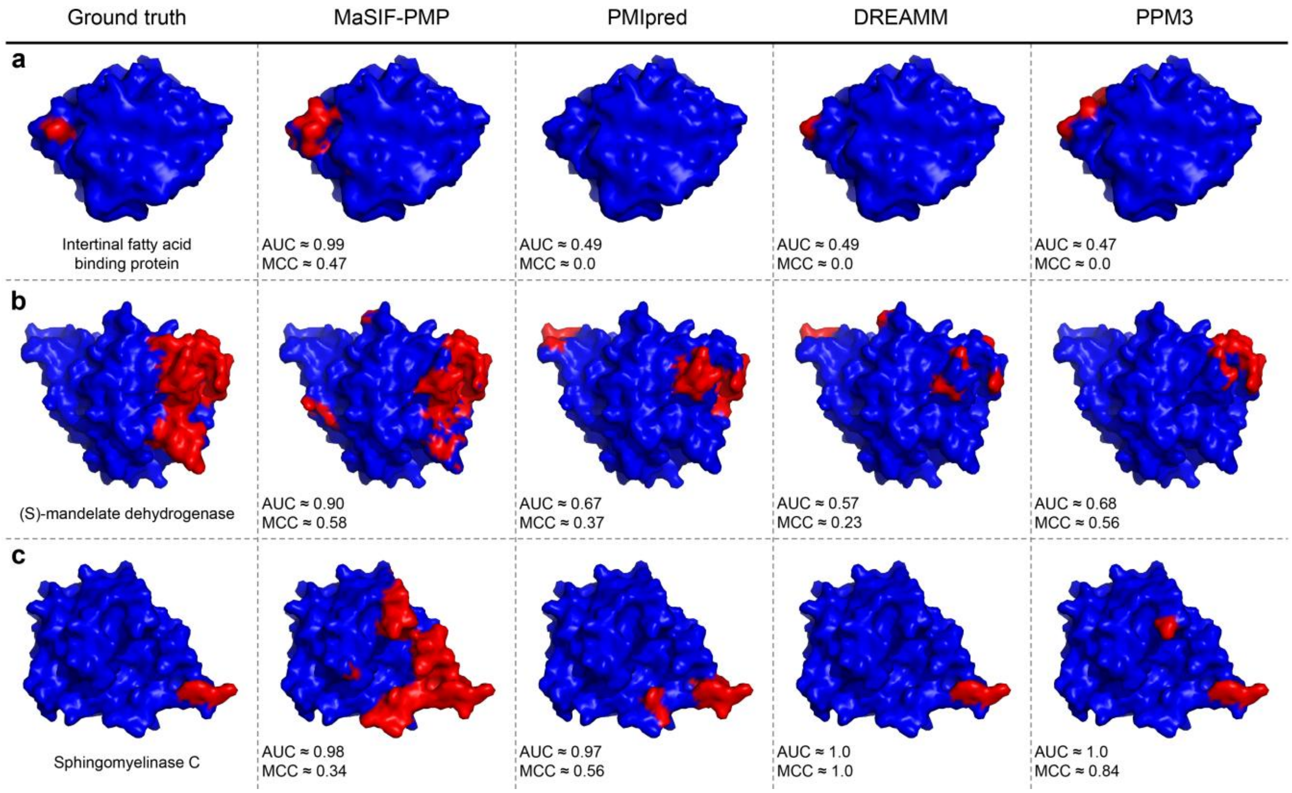

Supporting Figure 10. Visualization of ground-truth and predicted IBSs for three PMPs selected from the benchmark test set. Each column shows PMP molecular surfaces with colors indicating binary labels (blue for non-binding surface points or red for the IBS) for the ground-truth, MaSIF-PMP, PMIpred, DREAMM, and PPM3 columns. A threshold IBS score of 0.707 (Supporting Fig. 9) was applied to convert the continuous interface prediction scores for MaSIF-PMP into binary predictions. ROC AUC and MCC values were computed based on surface-level predictions for all three models. **(a)** Intestinal fatty acid binding protein (PDB ID: 3AKM). **(b)** (S)-mandelate dehydrogenase (PDB ID: 6BFG). **(c)** Sphingomyelinase C (PDB ID: 2DDR).

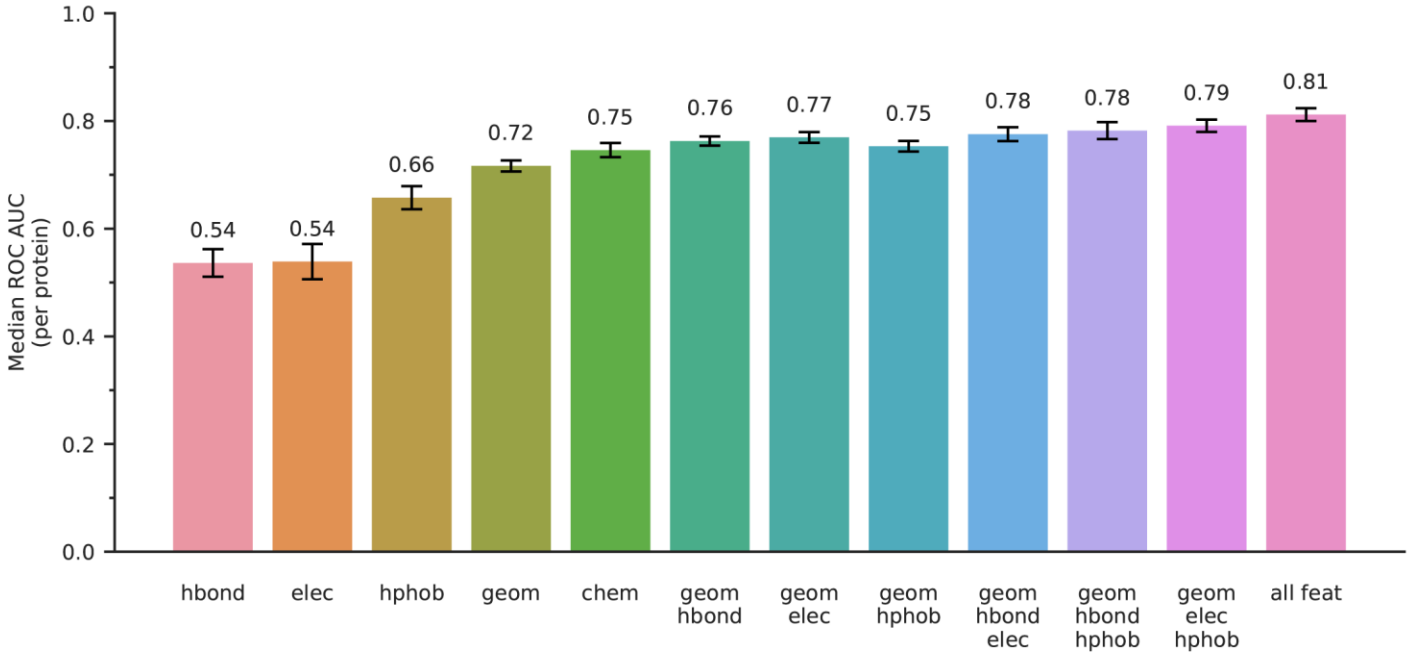

Supporting Figure 11. Ablation studies using 5-fold cross-validation with MaSIF-PMP trained on different subsets of surface features: location of free electrons/proton donors (hbond), Poisson–Boltzmann electrostatic potential (elec), hydropathy index (hphob), geometric features only (geom), chemical features only (chem), and all features combined (all feat). Combinations of geometric and chemical features are denoted using the corresponding abbreviations (*e.g.*, “geom hbond” indicates training on geometric features and hydrogen bond potential).

Analysis of surface features for IBS vs. non-IBS patches

We examined whether the distributions of surface features across all PMP surface patches of the dataset differed between interface and non-interface labels. In total, the dataset contains 5,918,254 surface patches, of which 763,986 are labeled as interface and 5,154,268 as non-interface. Patch-level feature values were computed by averaging the feature values of all vertices comprising each patch. The resulting distributions of surface feature values are shown in Supporting Figure 12. IBS patches exhibited slight rightward shifts in the distributions of two geometric features and hydrophobicity features relative to non-IBS patches. These results suggest that protrusion and hydrophobicity are important distinguishing characteristics, which is consistent with prior work by the Fuglebakk group.[18] In addition, the electrostatic potentials of IBS patches showed a modest rightward shift compared to non-IBS patches, in line with the general mechanisms of nonspecific protein–membrane interactions driven by electrostatic attraction between positively-charged protein surfaces and negatively charged lipid headgroups.

We further sought to determine the proportion of protruding and hydrophobic-protruding patches within IBS and non-IBS patches using multiple definitions based on the shape index (SI), distance-dependent curvature (DDC), and hydrophobicity features (see Supporting Table 5). We reasoned that protruding patches could be defined as those with positive values for patch-level geometric features, and hydrophobic-protruding patches as protruding additionally have positive hydrophobicity features. We acknowledge that the combination of curated surface features employed here may not fully recapitulate the "hydrophobic protrusion" definition proposed by the Fuglebakk group.[18] Nevertheless, the fraction of such patches was consistently higher for IBS than non-IBS patches, although the increase was not sufficiently pronounced to serve as a definitive criterion for IBS identification. Therefore, while hydrophobicity and geometric features representative of protrusions capture key structural tendencies of IBSs in a subset of PMPs, these results indicate that IBSs are characterized by subtle and complex combinations of multiple surface features, and that no single or pairwise feature is sufficient to reliably distinguish all interfaces from noninterface regions across the dataset.

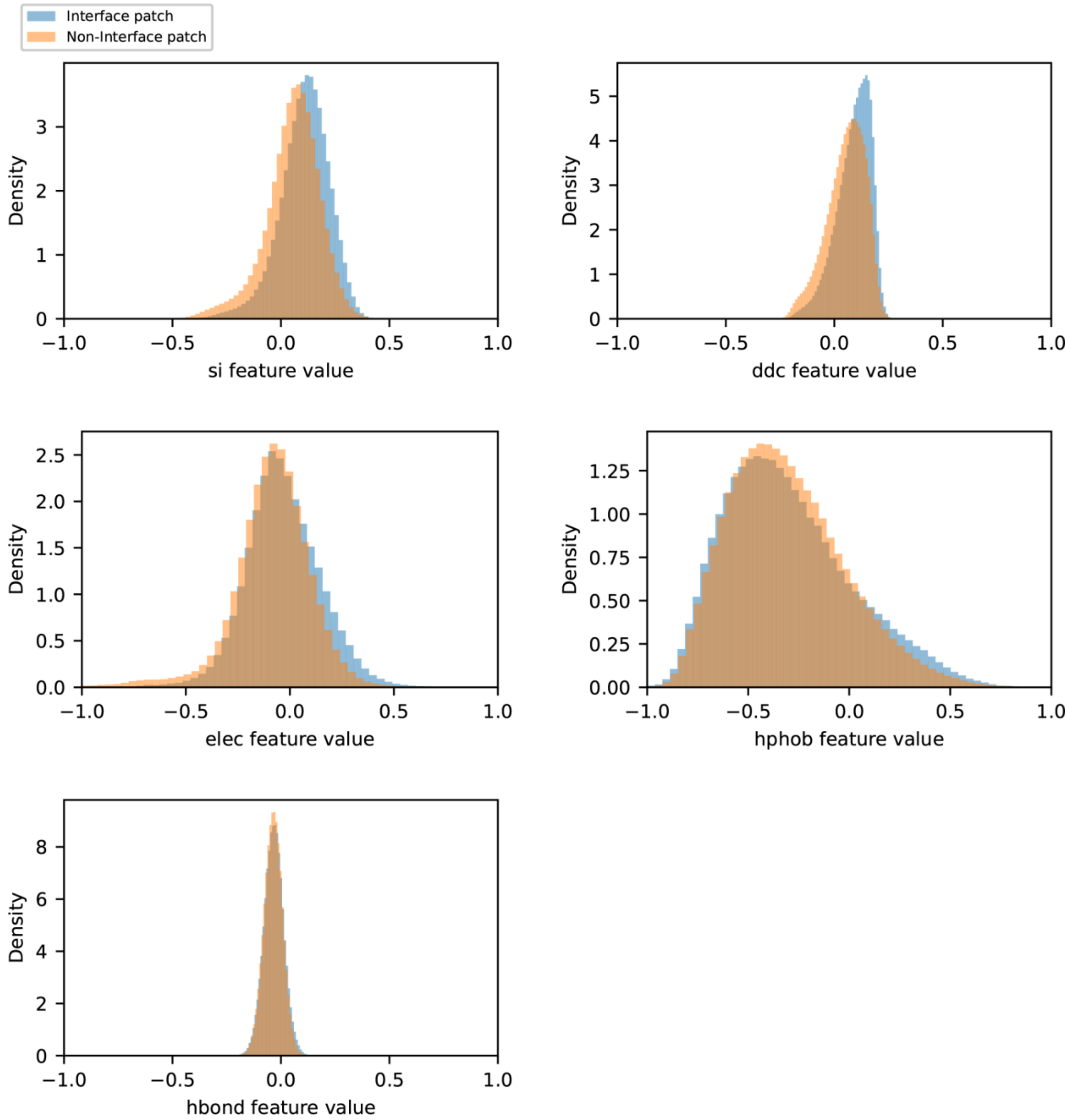

Supporting Figure 12. Distributions of surface features across all PMP surface patches in the dataset for interface and non-interface labels. 'si' denotes shape index, 'ddc' distance-dependent curvature, 'elec' electrostatic potential, 'hphob' hydropathy index, and 'hbond' free electrons/proton donors.

Supporting Table 5. Proportion of protruding ('Protrusion') and hydrophobic-protruding ('Hydrophobic protrusion') patches within interface and non-interface patches. Multiple definitions were used based on the shape index (SI), distance-dependent curvature (DDC), and hydrophobicity features. Percentages were calculated within each patch group, comprising 763,986 interface and 5,154,268 non-interface patches.

'Protrusion'

Definition	Percentage within interface patches	Percentage within non-interface patches
SI > 0	84.54%	71.25%
DDC > 0	85.94%	74.10%
SI > 0 & DDC > 0	79.88%	64.76%

'Hydrophobic protrusion'

Definition	Percentage within interface patches	Percentage within non-interface patches
SI > 0 & hydrophobicity > 0	13.99%	8.88%
SI > 0 & DDC > 0 & hydrophobicity > 0	13.37%	8.03%

Transfer learning of PPI-trained model to IBS predictions

We trained the MaSIF-site using the architecture, training set, and parameters used in the previous work.[3] For transfer learning strategy 1, we froze the parameters of the three convolutional layers and replaced the final multilayer perceptron (MLP) blocks with a deeper one: a fully connected network (FCN) of FC128, FC64, FC4, FC2. For transfer learning strategy 2, we froze the parameters of the three convolutional layers then added three new convolutional layers before the final MLP blocks. Both models were then trained for 50 epochs with all 5 surface features using the same soft grid parameters and data sets introduced in *Method* section for MaSIF-PMP. The schematics of model architectures of each transfer learning model are shown in Supporting Fig. 13.

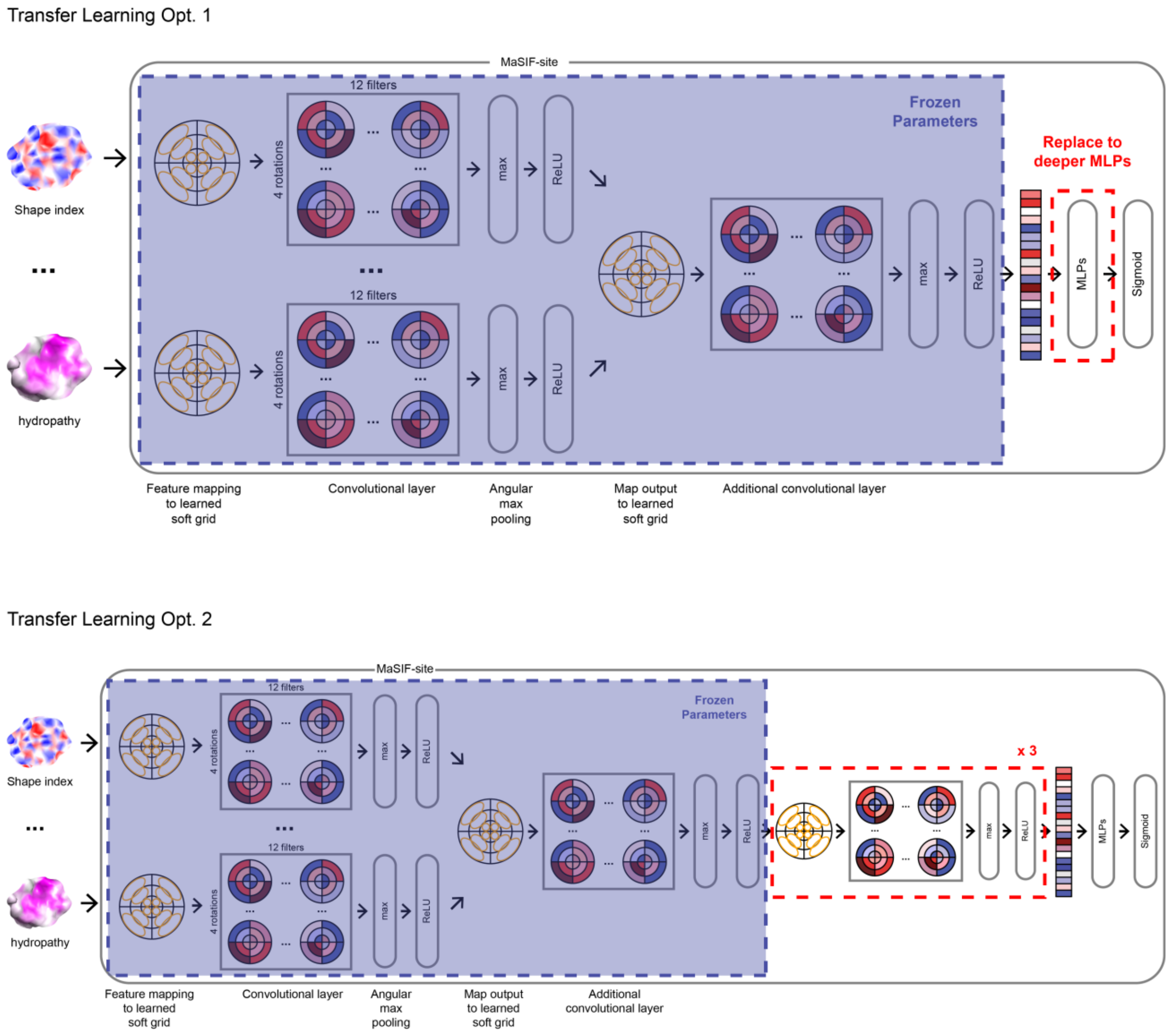

Supporting Figure 13. Network architectures of MaSIF-PMP with two different transfer learning strategies that derive models from the MaSIF-site model, which is trained for PPIs. The blue shaded regions represent frozen layers where parameters trained for PPIs are no longer updated.

Data augmentation using MD simulations of PMPs in aqueous solution

To circumvent complexity in preparing various membrane systems depending on PMPs and high computational cost of running simulations of those systems, we hypothesized that unbiased MD simulations of PMPs in aqueous solution may capture conformational dynamics (such as stochastic protrusions of lipid-binding residues) relevant to their membrane-bound states, thereby enabling efficient sampling of important conformations at a lower computational cost than simulating protein–membrane interactions. To test this hypothesis, we performed high-throughput MD[19] simulations of PMPs from the training set in aqueous solution, sampled representative conformations from trajectories, then augmented the training dataset with these MD-derived conformations to evaluate their impact on prediction for the test set.

High-throughput simulations of PMPs in aqueous solution were performed using high-throughput MD (HTMD).[19] From the training set, 503 proteins were selected for the simulations based on the absence of unmodeled or missing residues within the central regions of their sequences, as such gaps can result in trajectories with multiple disconnected segments freely diffusing in solution. Protein structures were protonated at pH 7.0, with standard charged termini (*i.e.*, N-terminus: $–NH_3^+$; C-terminus: $–COO^-$). Systems were solvated with TIP3P water and neutralized using Na^+ and $Cl^−$ ions at 0 M concentration. All systems were parameterized using the CHARMM36m force field.[20, 21] Each system underwent energy minimization using the conjugate gradient algorithm for 500 steps, followed by a 1 ns equilibration. Production simulations were then carried out for 10 ns under constant NPT conditions. The temperature was maintained at 298.15 K using a Langevin thermostat, and pressure was maintained at 1 bar using an isotropic Monte Carlo barostat. Simulation snapshots were saved every 50 ps during production. After completion, all trajectories were preprocessed using GROMACS 2021[22] to center and apply rotational and translational fitting of the protein.

Dynamic conformations of proteins sampled from simulations were clustered using CLoNe[23], an automated clustering algorithm based on principal component analysis (PCA) of the Cartesian coordinates of C_α atoms. The method first performs a nearest-neighbor step to estimate local densities for each data point, followed by identification of putative cluster centers as local density maxima. Clusters are merged, if necessary, using the Bhattacharyya coefficient[24], and outliers are removed via a Bayes classifier. CLoNe

requires only a single user-defined parameter, p_{dc}, which determines the number of clusters. The value of p_{dc} can be increased to reduce the number of clusters or decreased to increase it; integer values between 1 and 10 are generally sufficient, with many values yielding identical results. In this study, the default value of 4 was used for p_{dc}. After clustering protein conformations sampled from HTMD simulations using CLoNe, a total of 200 conformations were grouped into representative clusters of varying sizes. To ensure training on only "meaningful" conformational states, cluster centers were filtered based on cluster size, retaining only those containing at least 5% of the total simulation frames (≥10 frames) across the entire trajectory.

Ensemble learning with MaSIF-PMP and MD simulation data

We trained a version of the MaSIF-PMP network using an MD-augmented dataset, in which each protein was represented by multiple conformations sampled from HTMD simulations and clustered using the CLoNe algorithm. In this initial implementation, each conformation derived from a single PDB structure was treated as an independent input during training. No weighted averaging or feature aggregation across conformational ensembles was applied. As a result, the size of the training dataset increased from 1,059 to 1,896 protein structures. Training followed the same protocol as for the baseline MaSIF-PMP model. Training was performed for 100 epochs on an NVIDIA L40 GPU. The model was saved whenever the validation ROC AUC improved, with the final model checkpoint corresponding to epoch 61. No MD-based data augmentation was applied to proteins in the test set.

Despite the increased training data, the MD-augmented model exhibited similar predictive performance to the original model trained exclusively on crystal structures. On the test set, the MD-augmented network achieved a mean per-protein ROC AUC of 0.77 and a median of 0.78, compared to 0.76 (mean) and 0.78 (median) for the baseline model. This nearly identical performance indicates that conformations sampled from aqueous-phase simulations provide limited improvement to IBS predictions, and thus this strategy was not pursued further in favor of alternative MD simulations of PMP–membrane interactions.

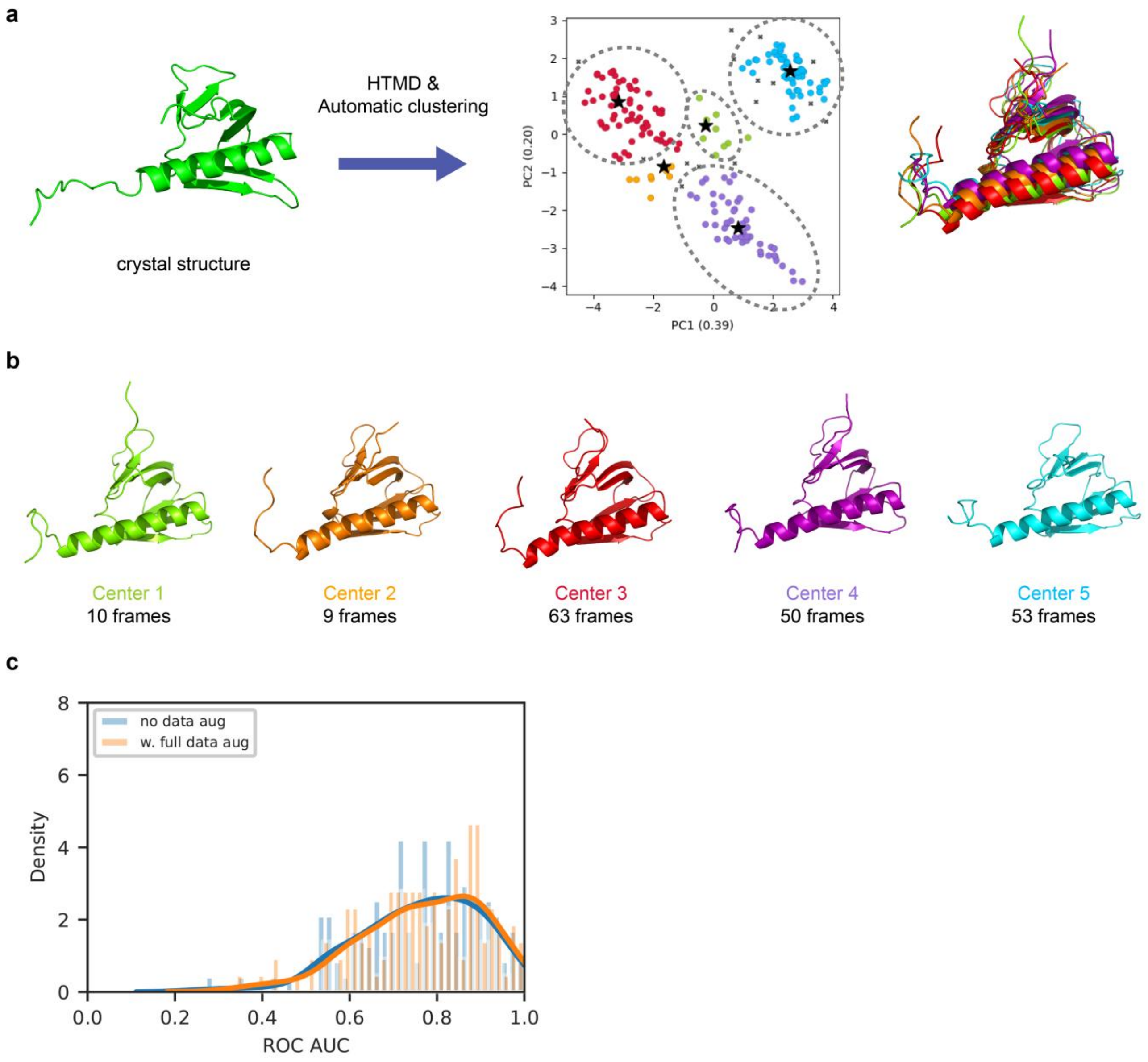

Supporting Figure 14. Data augmentation using MD simulations. **(a)** Schematic overview of the workflow and concept for using MD simulations to capture protein conformational dynamics. All snapshots and plots are based on G-protein coupled receptor kinase 2 (PDB ID: 1BAK). Gray dashed circles highlight representative cluster centers selected from the trajectory based on frame counts. The ensemble snapshot on the right shows the superposition of cluster center structures. **(b)** Representative cluster centers sampled from the HTMD trajectory using the CLoNe clustering algorithm. **(c)** Distribution of per-protein surface-level ROC AUC scores across the test set, predicted by MaSIF-PMP models trained with and without MD-based data augmentation. Kernel density estimates were generated using Gaussian kernels.

Case studies using HMMM simulations

α-tocopherol transfer protein (α-TTP)

HMMM simulations of α-tocopherol transfer protein (α-TTP) were conducted using the crystal structure from PDB ID: 3W67, chain A. Simulations were performed with α-TTP in complex with α-tocopherol (α-Tol) because α-TTP binds to membranes in its ligand-bound state and undergoes a ligand-exchange mechanism between phosphatidylinositol phosphates (PIPs) and α-Tol.[25] Although α-Tol is buried within the hydrophobic core of the protein and has minimal effect on the molecular surface, it was included to accurately model the biologically relevant conformation.

Membrane binding in α-TTP occurs primarily via direct interactions between the negatively charged PIP_2 headgroups and basic residues located near the opening of the ligand-binding cavity.[25] The HMMM membrane system was constructed to replicate the lipid composition used in prior MD study[25] of α-TTP, consisting of 60 DOPC, 40 DOPE, and 2 PI(4,5)P_2 molecules per leaflet, with DCLE molecules modeling the hydrophobic core (Supporting Table 6). For replica simulations (Supporting Fig. 15a), we tested three systems with different orientations of α-TTP with respect to the HMMM membrane: the same orientation of structure from RCSB PDB (*ori.1*), rotated along the *x* axis by 90° (*ori.2*), and rotated along the *y* axis by 90° (*ori. 3*). We performed three replica simulations with different initial velocities for each orientation for a total of 9 independent simulations. Detailed system information is provided in Supporting Table 6.

All systems were energy-minimized using the steepest descent algorithm until the maximum force between atoms reached the criterion of <1000 kJ mol^{-1} nm^{-2}. After energy minimization, systems were equilibrated at constant NVT for 250 ps then further equilibrated for 1,625 ps at constant NPT. Positional restraints on HMMM lipid atoms were gradually reduced during equilibration. Production simulations were performed for 100 ns under NPT conditions at 300 K using a velocity-rescale thermostat (time constant = 1.0 ps) and at 1 bar using a semi-isotropic stochastic cell-rescaling barostat (time constant = 5.0 ps, compressibility = 4.5×10^{-5} bar^{-1}). All MD simulations were conducted with a 2 fs timestep using the leapfrog integrator in GROMACS 2021.[22] Verlet lists were generated with a 1.2 nm neighbor list cutoff, van der Waals interactions were modeled using a Lennard-Jones potential with a 1.2 nm cutoff that was smoothly shifted to zero between 1.0 and 1.2 nm, and electrostatic interactions were calculated using the smooth

particle-mesh Ewald method with a short-range cutoff of 1.2 nm.[26] Bonds involving hydrogen atoms were constrained using the LINCS algorithm.[27] Further simulation parameters and raw data are available in the published dataset (DOI: 10.5061/dryad.1rn8pk175). α-TTP exhibited stable membrane interactions only in orientation 1, with no consensus IBSs detected for other orientations (Supporting Fig. 15); consensus IBSs are defined in the next section.

Supporting Table 6. Number of components for simulated α-TTP systems.

		ori.1	*ori.2*	*ori.3*
α-TTP		1	1	1
Lipid molecules	$PI(4,5)P_2$	4	4	4
	PE	80	80	80
	PC	120	120	120
	DCLE	1,192	1,196	1,192
α-Tol		1	1	1
Water molecules		23,202	21,528	22,331
Na^+ ions		14	14	14
Cl^- ions		0	0	0
Total atoms		97,130	92,140	94,517

Defining consensus and union IBS label based on trajectories of replica simulations

Using replica simulations with multiple protein orientations relative to the membrane, we defined consensus IBS labels based on the fraction of simulation time during which protein–membrane contacts were observed. The final 10 ns of each replica were used to define these labels because stable contacts were consistently observed in this interval. Consensus IBS regions were designated based on the residues within 0.5 nm of any lipid (except the DCLE molecules that escaped from the bilayer) atom for at least 90% of the aggregated production phases (Supporting Fig. 15b). Union IBS labels were defined by combining consensus IBS labels for each orientation (Supporting Fig. 15c).

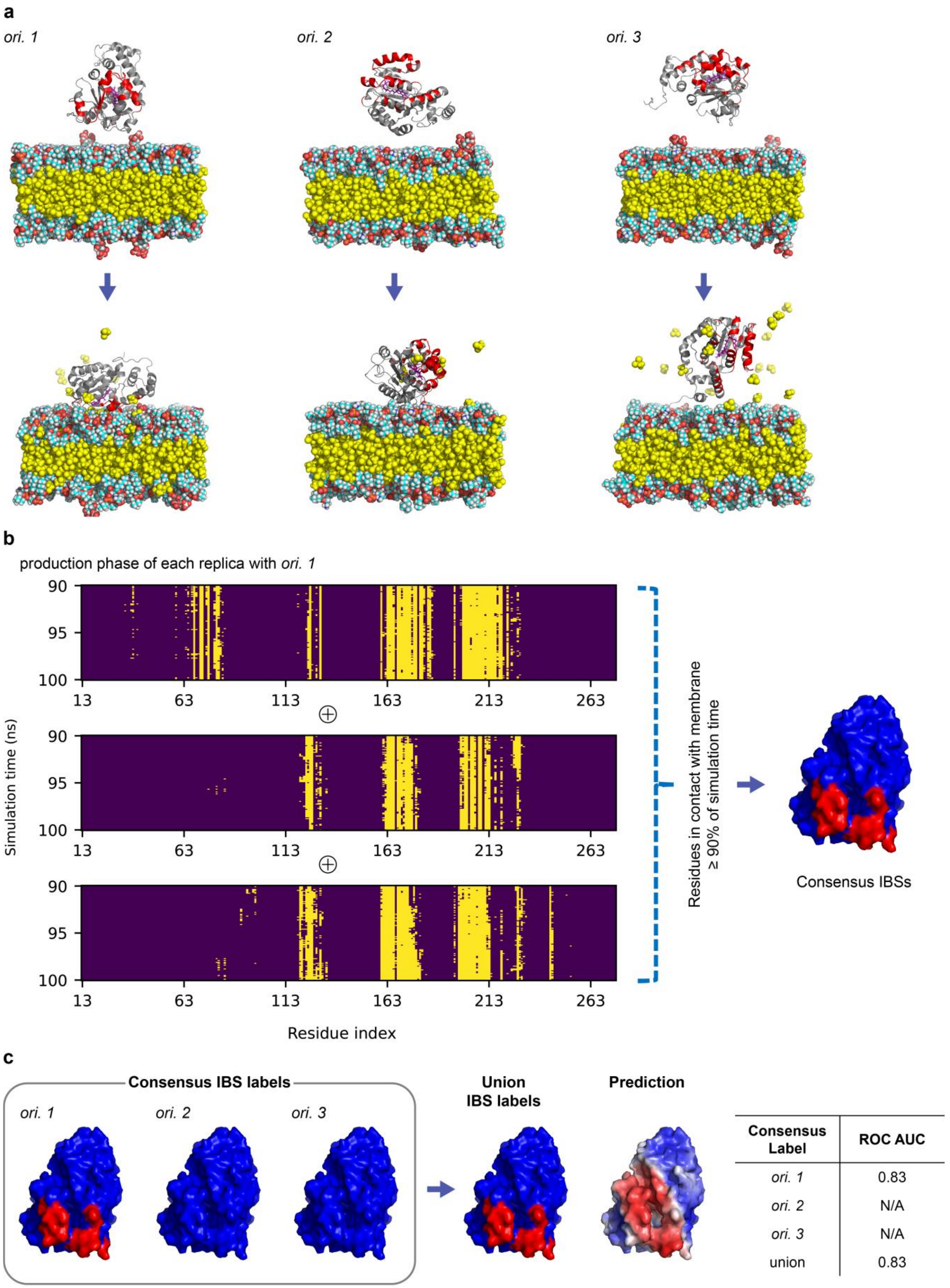

Consensus Label	ROC AUC
ori. 1	0.83
ori. 2	N/A
ori. 3	N/A
union	0.83

Supporting Figure 15. Replica simulations of α-TTP to check the consistency and robustness of IBS labels determined based on HMMM MD. Replica simulations were performed with α-TTP in multiple orientations with respect to HMMM membrane. **(a)** Representative snapshots of α-TTP before/after 100 ns HMMM MD simulations across multiple initial orientations (*ori.*). Snapshots of replica 1 for each orientation are presented. The IBS region derived from replica 1 of orientation 1 is highlighted in red for comparison between simulations with different orientations. The HMMM membrane is shown in a van der Waals representation; carbon atoms in cyan, hydrogen atoms in white, oxygen atoms in red, nitrogen atoms in blue, phosphorus atoms in orange, α-tocopherol in purple, and organic solvent 1,1-dichloroethane (DCLE) in yellow. Water molecules and ions are not shown to aid visualization. **(b)** Defining consensus IBSs based on the fraction of simulation time in which protein–membrane contacts were observed. The final 10 ns of each replica were considered as the production phase, and we applied a 90% cutoff to designate consensus IBS regions. Only results from orientation 1 (*ori.1*) are shown in the figure. For the barcode plots, purple indicates no contact while yellow indicates contact between the protein and membrane. **(c)** Snapshots of consensus IBS labels for each orientation, their union, and predicted IBS scores for α-TTP. Tables show corresponding ROC AUC values computed with different ground-truth label types.

Oxysterol-binding protein homologue (Osh4)

HMMM simulation of oxysterol-binding protein homologue (Osh4) was performed using the crystal structure from PDB ID: 1ZHZ (chain A). Osh4 is known to undergo conformational rearrangement when interacting with trans-Golgi network (TGN) anionic membrane. A TGN membrane was prepared using the same lipid compositions as in a prior MD study of Osh4 (Supporting Table 7).[28] The system was neutralized with NaCl counterions.

MD simulations were performed for 100 ns under NPT conditions at 303.15 K and 1 bar, using the velocity-rescale thermostat and semi-isotropic stochastic cell-rescaling barostat. The same parameters as in the simulation of α-TTP were used for Osh4 simulations. Further simulation parameters and raw data are available in the published dataset (DOI: 10.5061/dryad.1rn8pk175). For the ROC AUC computation, we also compared HMMM labels to ground-truth labels derived from previous MD studies that reported six separated membrane-binding domains (referred to as the “paper” labels in Supporting Fig. 16).[28, 29]

Supporting Table 7. Composition of simulated system for the oxysterol-binding protein homologue (Osh4). Acronyms: TGN, trans-Golgi network; ERG, ergosterol; DYPC, PC(16:1(9Z)/16:1(9Z)); DYPE, PE(16:1(9Z)/16:1(9Z)); POPA, PA(16:0/18:1(9Z)); POPI, PI(16:0/18:1(9Z)); POPS, PS(16:0/18:1(9Z)); PYPE, PE(16:0/16:1(9Z)); PYPI, PI(16:0/16:1(9Z)); YOPC, PC(16:1(9Z)/18:1(9Z)); YOPE, PE(16:1(9Z)/18:1(9Z)); DCLE, 1,1-dichloroethane. For lipid names, the first two characters indicate the headgroup type, while the numbers and "Z" notation denote the acyl chain length and position of the cis double bond, respectively.

		Number
Osh4		1
TGN	ERG	36
	DYPC	58
	DYPE	12
	POPA	8
	POPI	54
	POPS	10
	PYPE	8
	PYPI	56
	YOPC	46
	YOPE	12
	DCLE	1,376
Water molecules		36,565
Na^+ ions		138
Cl^- ions		0
Total atoms		148,753

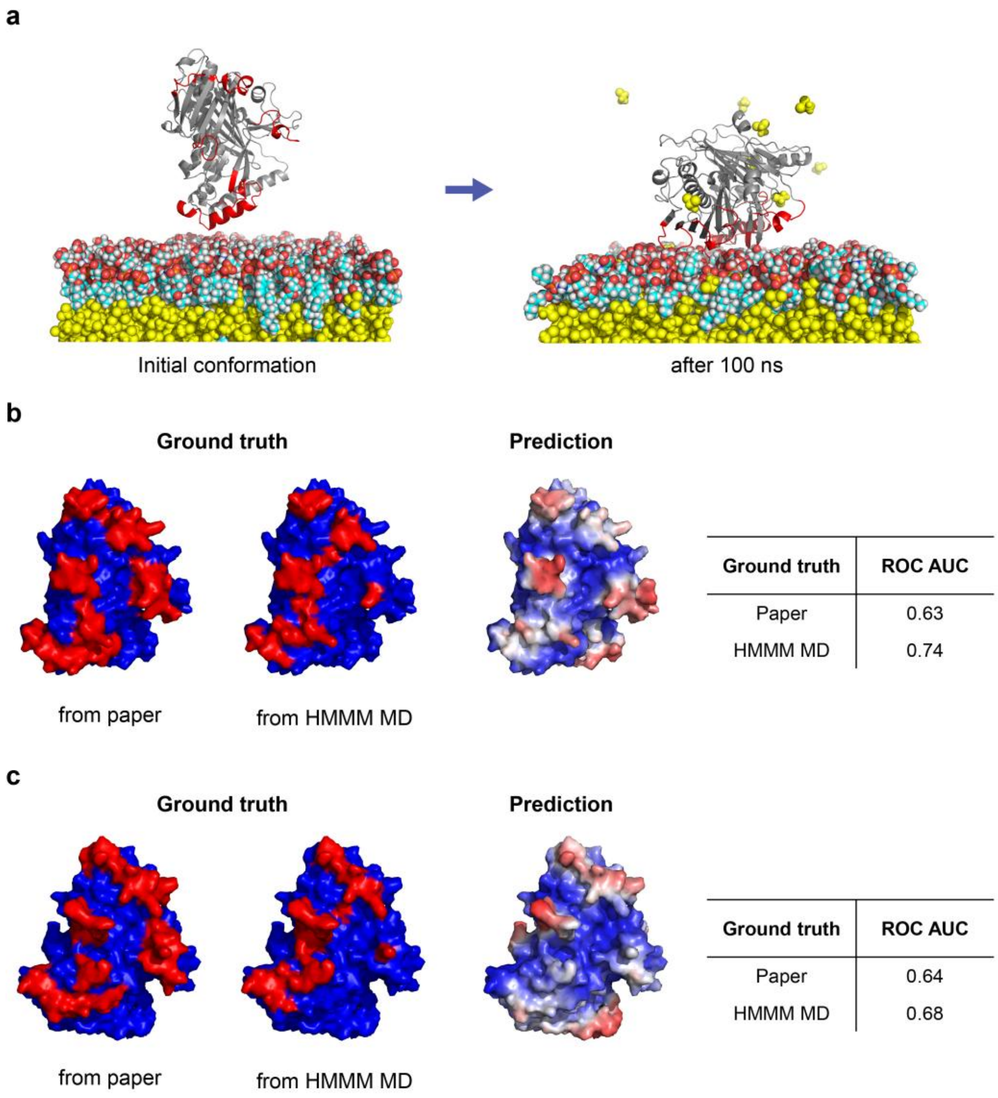

Ground truth	ROC AUC
Paper	0.63
HMMM MD	0.74

Ground truth	ROC AUC
Paper	0.64
HMMM MD	0.68

Supporting Figure 16. Case study with Osh4 using HMMM MD simulations. **(a)** Snapshot of Osh4 (PDB ID: 1ZHZ) interacting with the trans-Golgi network (TGN) membrane, sampled from HMMM MD simulations. Ground-truth membrane-binding interfaces from prior studies[28, 29] are highlighted in red. Color schemes follow the same definition used in Supporting Figure 15a. **(b)** MaSIF-PMP prediction on the crystal structure of Osh4. Reported ROC AUC scores correspond to surface-level predictions. ROC AUC values are computed using ground-truth labels from either previous studies (referred to as the “paper” ground-truth labels) or from the HMMM MD simulations. **(c)** MaSIF-PMP prediction on the membrane-bound conformation of Osh4 captured from HMMM simulations. Reported ROC AUC values are also based on surface-level predictions.

Representative HMMM membrane types: anionic and zwitterionic

To investigate membrane-binding behaviors of PMPs with poor MaSIF-PMP prediction performance, we selected two representative membrane types: anionic and zwitterionic. These types reflect distinct chemical characteristics commonly observed in biological systems, such as of the trans-Golgi network (TGN) and endoplasmic reticulum (ER), two well-studied membranes known for their differing lipid compositions and surface chemistries.[30] Although both biological membranes consist of a mix of zwitterionic and anionic lipids, we simplified the model systems by including only zwitterionic phosphatidylethanolamine (PE) and anionic phosphatidylserine (PS). This simplification was guided by the widespread biological relevance of PE and PS, as well as their comparable lengths, which help minimize geometric variations in the membrane surface. Accordingly, the HMMM membranes were prepared using the following lipid ratios: PE:PS = 100:0 for the zwitterionic membrane, and PE:PS = 50:50 for the anionic membrane. For the replica simulations, three initial protein orientations with respect to the HMMM membrane were tested, identical to those used for the α-TTP systems. A single replica simulation was performed for each orientation.

The structure of phospholipase A2 was obtained from PDB ID: 1OZY. Additional disulfide bonds were modeled between the following cysteine pairs: C11-C77, C29-C45, C44-C105, C51-C98, C61-C91, and C84-C96. Detailed system information is provided in Supporting Table 8. Further simulation parameters and raw data are available in the published dataset (DOI: 10.5061/dryad.1rn8pk175).

The structure of glycosyl hydrolase was obtained from PDB ID: 4LPL. As the net charge of the glycosyl hydrolase system with the zwitterionic HMMM membrane was zero, the system was neutralized using 0.02 M NaCl. Detailed system information is provided in Supporting Table 8. Further simulation parameters and raw data are available in the published dataset (DOI: 10.5061/dryad.1rn8pk175).

We defined the consensus IBS labels of the two proteins with either HMMM membrane types in the same manner used for α-TTP system in the previous section. We ran replica simulations for each initial orientation, used the final 10 ns of each replica as the production phase, and applied a time-fraction cutoff of 95% to define the consensus IBS labels for each orientation. The union of the HMMM-derived IBS labels for each replica was compared with the MaSIF-PMP predictions to compute the ROC AUC values reported in the main text.

Supporting Table 8. Number of components for each simulated system. The final composition of each system is summarized below. PE denotes phosphatidylethanolamine, PS denotes phosphatidylserine, and DCLE refers to the organic solvent 1,1-dichloroethane.

			PE	PS	DCLE	Water molecules	Na^+ ions	Cl^- ions	Total atoms
Phospholipase A2	Anionic membrane	*ori.1*	100	100	1,008	19,157	99	0	79,870
		ori.2	100	100	1,008	18,664	99	0	78,391
		ori.3	100	100	986	16,934	99	0	73,025
	Zwitterionic membrane	*ori.1*	200	0	1,004	19,079	0	1	79,306
		ori.2	200	0	1,000	18,562	0	1	77,723
		ori.3	200	0	1,004	16,837	0	1	72,580
Glycosyl hydrolase	Anionic membrane	*ori.1*	100	100	998	18,026	100	0	76,842
		ori.2	100	100	1,008	17,985	100	0	76,799
		ori.3	100	100	998	17,755	100	0	76,029
	Zwitterionic membrane	*ori.1*	200	0	1,004	17,914	6	6	76,266
		ori.2	200	0	1,004	17,924	6	6	76,296
		ori.3	200	0	991	17,674	6	6	75,442

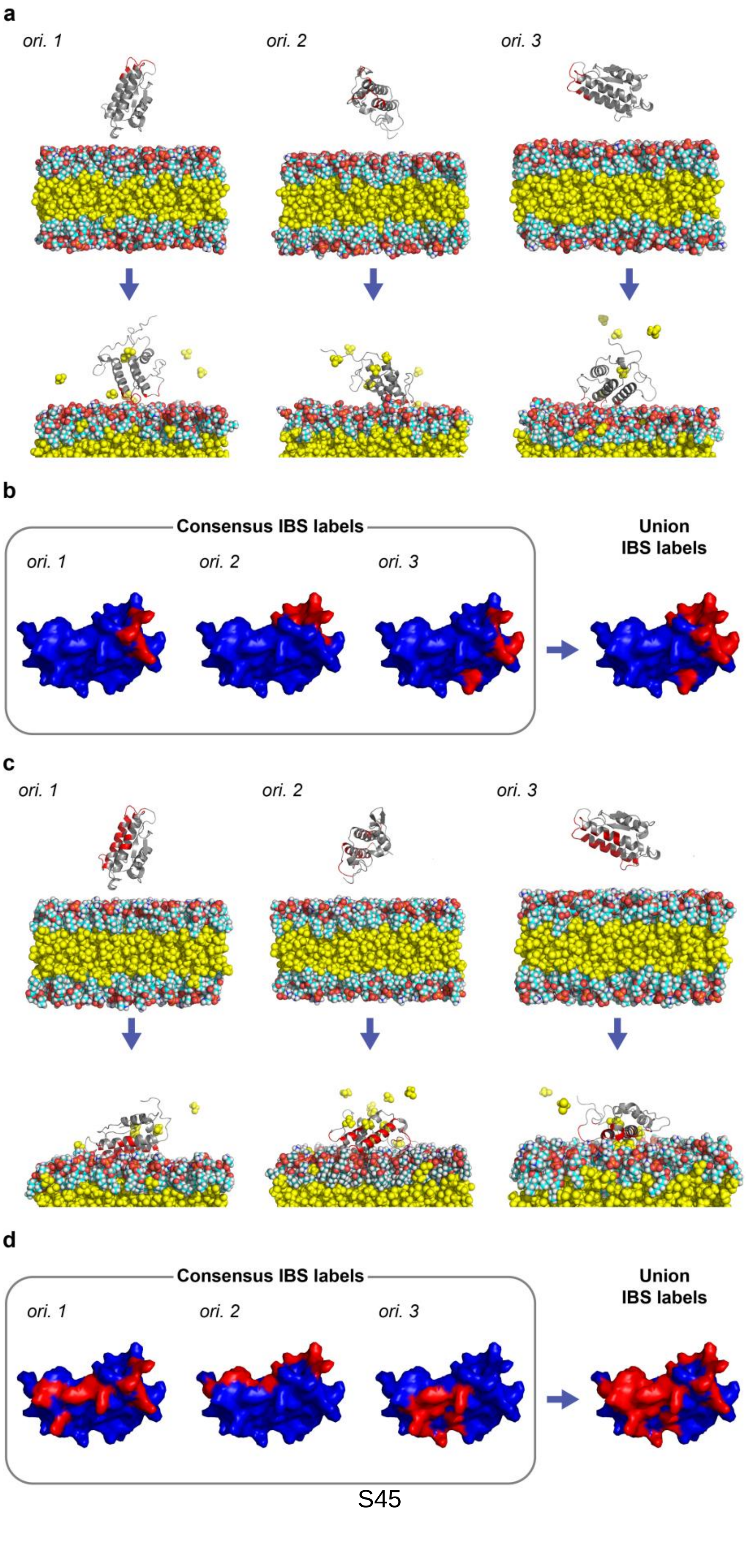
a
ori. 1
ori. 2
ori. 3
b
Consensus IBS labels
ori. 1
ori. 2
ori. 3
Union
IBS labels
c
ori. 1
ori. 2
ori. 3
d
Consensus IBS labels
ori. 1
ori. 2
ori. 3
Union
IBS labels

Supporting Figure 17. Replica simulations of the phospholipase A2 (PDB ID: 1OZY) system to check the consistency and robustness in IBS labels determined based on HMMM MD. Replica simulations were performed with the protein initialized in multiple orientations with respect to HMMM membrane. **(a)** Representative snapshots of phospholipase A2 after 100 ns HMMM MD simulation with the anionic membrane across multiple initial orientations (*ori.*). IBS regions derived from replica 1 (*ori.1*) is highlighted in red for comparison across orientations. Color schemes follow the same definition used in Supporting Figure 15a. **(b)** Snapshots of consensus IBS labels for each orientation and their union derived from HMMM simulations with the anionic membrane. Consensus IBSs were defined based on the fraction of simulation time in which protein–membrane contacts were observed, using the final 10 ns of each replica as the production phase and a 95% cutoff for designation. **(c)** Representative snapshots of phospholipase A2 after 100 ns of HMMM MD with the zwitterionic membrane, and **(d)** the corresponding consensus IBS labels for each orientation and their union, presented in the same manner as panels (a) and (b).

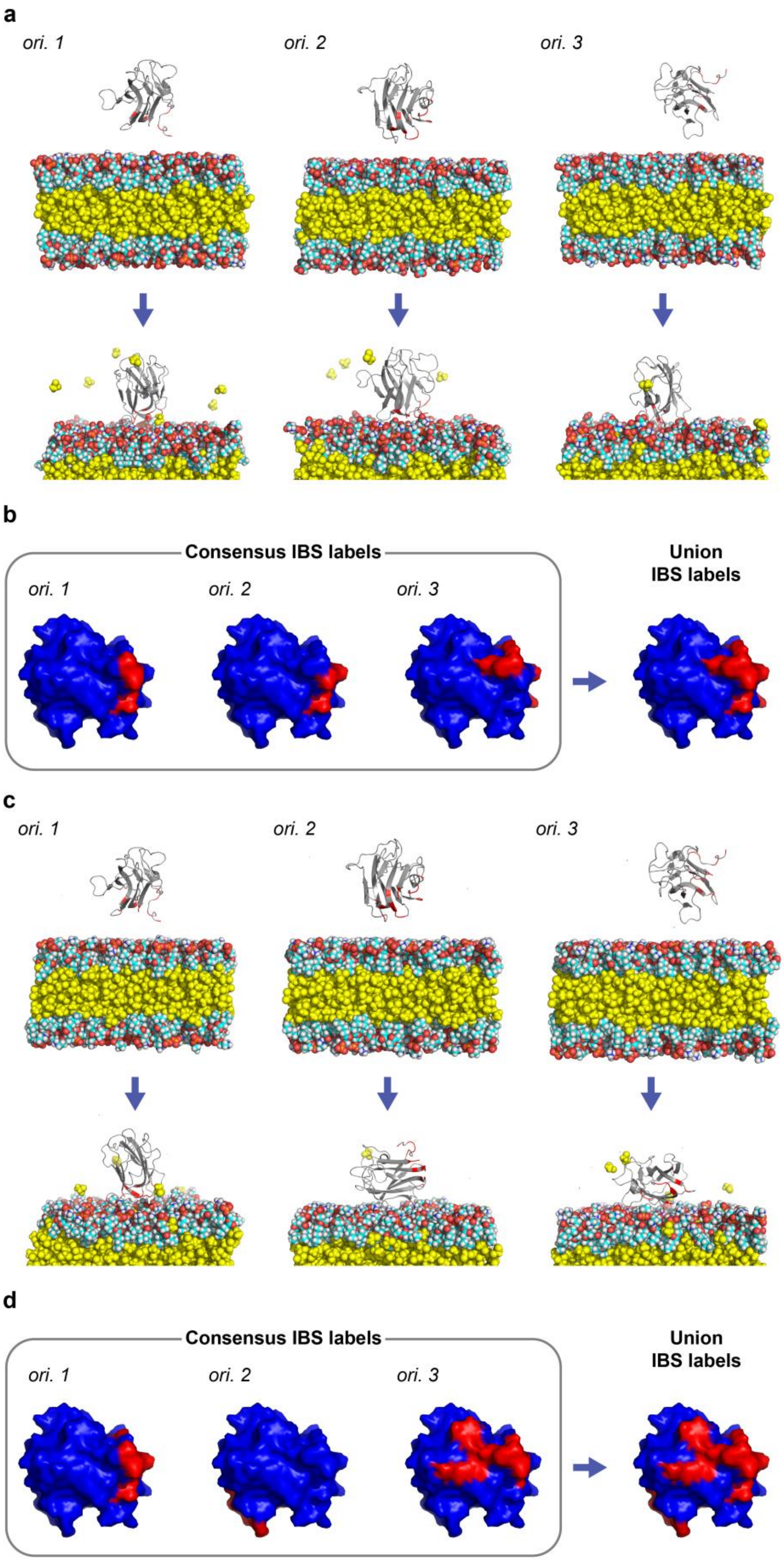
a
ori. 1
ori. 2
ori. 3
b
Consensus IBS labels
ori. 1
ori. 2
ori. 3
Union
IBS labels
c
ori. 1
ori. 2
ori. 3
d
Consensus IBS labels
ori. 1
ori. 2
ori. 3
Union
IBS labels

Supporting Figure 18. Replica simulations of the glycosyl hydrolase (PDB ID: 4LPL) system to check the consistency and robustness in IBS labels determined based on HMMM MD. Replica simulations were performed with the protein initialized in multiple orientations with respect to HMMM membrane. **(a)** Representative snapshots and **(b)** corresponding consensus IBS labels from 100 ns HMMM MD simulations with the anionic membrane, presented in the same manner as Supporting Fig. 17a-b. **(c)** Equivalent snapshots and **(d)** consensus IBS labels from simulations with the zwitterionic membrane.

Supporting References

(1) Tubiana, T.; Sillitoe, I.; Orengo, C.; Reuter, N. Dissecting peripheral protein-membrane interfaces. *PLOS Computational Biology* **2022**, *18* (12), e1010346. DOI: 10.1371/journal.pcbi.1010346.

(2) Sillitoe, I.; Bordin, N.; Dawson, N.; Waman, V. P.; Ashford, P.; Scholes, H. M.; Pang, C. S. M.; Woodridge, L.; Rauer, C.; Sen, N. CATH: increased structural coverage of functional space. *Nucleic acids research* **2021**, *49* (D1), D266-D273.

(3) Gainza, P.; Sverrisson, F.; Monti, F.; Rodolà, E.; Boscaini, D.; Bronstein, M. M.; Correia, B. E. Deciphering interaction fingerprints from protein molecular surfaces using geometric deep learning. *Nature Methods* **2020**, *17* (2), 184-192. DOI: 10.1038/s41592-019-0666-6.

(4) Sanner, M. F.; Olson, A. J.; Spehner, J. C. Reduced surface: an efficient way to compute molecular surfaces. *Biopolymers* **1996**, *38* (3), 305-320.

(5) Yin, S.; Proctor, E. A.; Lugovskoy, A. A.; Dokholyan, N. V. Fast screening of protein surfaces using geometric invariant fingerprints. *Proc Natl Acad Sci U S A* **2009**, *106* (39), 16622-16626. DOI: 10.1073/pnas.0906146106 From NLM.

(6) Zhou, Q. Pymesh—geometry processing library for python. *Software available for download at https://github. com/PyMesh/PyMesh* **2019**, *7*.

(7) Dolinsky, T. J.; Czodrowski, P.; Li, H.; Nielsen, J. E.; Jensen, J. H.; Klebe, G.; Baker, N. A. PDB2PQR: expanding and upgrading automated preparation of biomolecular structures for molecular simulations. *Nucleic acids research* **2007**, *35* (suppl_2), W522-W525.

(8) Baker, N. A.; Sept, D.; Joseph, S.; Holst, M. J.; McCammon, J. A. Electrostatics of nanosystems: application to microtubules and the ribosome. *Proceedings of the National Academy of Sciences* **2001**, *98* (18), 10037-10041.

(9) Kortemme, T.; Morozov, A. V.; Baker, D. An Orientation-dependent Hydrogen Bonding Potential Improves Prediction of Specificity and Structure for Proteins and Protein–Protein Complexes. *Journal of Molecular Biology* **2003**, *326* (4), 1239-1259. DOI: 10.1016/s0022-2836(03)00021-4.

(10) Kyte, J.; Doolittle, R. F. A simple method for displaying the hydropathic character of a protein. *J Mol Biol* **1982**, *157* (1), 105-132. DOI: 10.1016/0022-2836(82)90515-0 From NLM.

(11) Monti, F.; Boscaini, D.; Masci, J.; Rodola, E.; Svoboda, J.; Bronstein, M. M. Geometric Deep Learning on Graphs and Manifolds Using Mixture Model CNNs. In *IEEE Conference on Computer Vision and Pattern Recognition (CVPR)*, 2017, 2017; pp 5115-5124.

(12) Masci, J.; Boscaini, D.; Bronstein, M.; Vandergheynst, P. Geodesic convolutional neural networks on riemannian manifolds. In *Proceedings of the IEEE international conference on computer vision workshops*, 2015, 2015; pp 37-45.

(13) Krizhevsky, A.; Sutskever, I.; Hinton, G. E. Imagenet classification with deep convolutional neural networks. *Advances in neural information processing systems* **2012**, *25*.

(14) van Hilten, N.; Verwei, N.; Methorst, J.; Nase, C.; Bernatavicius, A.; Risselada, H. J. PMIpred: a physics-informed web server for quantitative protein–membrane interaction prediction. *Bioinformatics* **2024**, *40* (2), btae069.

(15) Chatzigoulas, A.; Cournia, Z. Predicting protein–membrane interfaces of peripheral membrane proteins using ensemble machine learning. *Briefings in Bioinformatics* **2022**, *23* (2). DOI: 10.1093/bib/bbab518 (accessed 7/3/2023).

(16) Lomize, A. L.; Todd, S. C.; Pogozheva, I. D. Spatial arrangement of proteins in planar and curved membranes by PPM 3.0. *Protein Science* **2022**, *31* (1), 209-220.

(17) Porollo, A.; Meller, J. Prediction-based fingerprints of protein-protein interactions. *Proteins* **2007**, *66* (3), 630-645. DOI: 10.1002/prot.21248 From NLM.

(18) Fuglebakk, E.; Reuter, N. A model for hydrophobic protrusions on peripheral membrane proteins. *PLoS computational biology* **2018**, *14* (7), e1006325.

(19) Doerr, S.; Harvey, M. J.; Noé, F.; De Fabritiis, G. HTMD: High-Throughput Molecular Dynamics for Molecular Discovery. *Journal of Chemical Theory and Computation* **2016**, *12* (4), 1845-1852. DOI: 10.1021/acs.jctc.6b00049.

(20) Best, R. B.; Zhu, X.; Shim, J.; Lopes, P. E. M.; Mittal, J.; Feig, M.; MacKerell, A. D., Jr. Optimization of the Additive CHARMM All-Atom Protein Force Field Targeting Improved Sampling of the Backbone ϕ, ψ and Side-Chain χ1 and χ2 Dihedral Angles. *Journal of Chemical Theory and Computation* **2012**, *8* (9), 3257-3273. DOI: 10.1021/ct300400x.

(21) Huang, J.; Rauscher, S.; Nawrocki, G.; Ran, T.; Feig, M.; De Groot, B. L.; Grubmüller, H.; MacKerell Jr, A. D. CHARMM36m: an improved force field for folded and intrinsically disordered proteins. *Nature methods* **2017**, *14* (1), 71-73.

(22) Abraham, M. J.; Murtola, T.; Schulz, R.; Páll, S.; Smith, J. C.; Hess, B.; Lindahl, E. GROMACS: High performance molecular simulations through multi-level parallelism from laptops to supercomputers. *SoftwareX* **2015**, *1*, 19-25.

(23) Träger, S.; Tamò, G.; Aydin, D.; Fonti, G.; Audagnotto, M.; Dal Peraro, M. CLoNe: automated clustering based on local density neighborhoods for application to biomolecular structural ensembles. *Bioinformatics* **2021**, *37* (7), 921-928. DOI: 10.1093/bioinformatics/btaa742 From NLM.

(24) Bhattacharyya, A. On a measure of divergence between two statistical populations defined by their probability distribution. *Bulletin of the Calcutta Mathematical Society* **1943**, *35*, 99-110.

(25) Lamprakis, C.; Stocker, A.; Cascella, M. Mechanisms of recognition and binding of α-TTP to the plasma membrane by multi-scale molecular dynamics simulations. *Frontiers in molecular biosciences* **2015**, *2*, 36.

(26) Essmann, U.; Perera, L.; Berkowitz, M. L.; Darden, T.; Lee, H.; Pedersen, L. G. A smooth particle mesh Ewald method. *The Journal of Chemical Physics* **1995**, *103* (19), 8577-8593. DOI: 10.1063/1.470117 (accessed 2023-11-17T23:28:50).

(27) Hess, B.; Bekker, H.; Berendsen, H. J. C.; Fraaije, J. G. E. M. LINCS: A linear constraint solver for molecular simulations. *Journal of Computational Chemistry* **1997**, *18* (12), 1463-1472. DOI: 10.1002/(sici)1096-987x(199709)18:12<1463::aid-jcc4>3.0.co;2-h (accessed 2023-11-17T23:45:07).

(28) Monje-Galvan, V.; Klauda, J. B. Peripheral membrane proteins: Tying the knot between experiment and computation. *Biochim Biophys Acta* **2016**, *1858* (7 Pt B), 1584-1593. DOI: 10.1016/j.bbamem.2016.02.018 From NLM.

(29) Rogaski, B.; Klauda, J. B. Membrane-binding mechanism of a peripheral membrane protein through microsecond molecular dynamics simulations. *Journal of molecular biology* **2012**, *423* (5), 847-861.

(30) Monje-Galvan, V.; Klauda, J. B. Modeling yeast organelle membranes and how lipid diversity influences bilayer properties. *Biochemistry* **2015**, *54* (45), 6852-6861.
